# Recent Advances in Paper-Based Electronics: Emphasis on Field-Effect Transistors and Sensors

**DOI:** 10.3390/bios15050324

**Published:** 2025-05-19

**Authors:** Dimitris Barmpakos, Apostolos Apostolakis, Fadi Jaber, Konstantinos Aidinis, Grigoris Kaltsas

**Affiliations:** 1 microSENSES Laboratory, Department of Electrical and Electronics Engineering, University of West Attica, 12244 Athens, Greece; apapostolakis@uniwa.gr (A.A.); g.kaltsas@uniwa.gr (G.K.); 2 Department of Biomedical Engineering, Ajman University, Ajman P.O. Box 346, United Arab Emirates; f.jaber@ajman.ac.ae; 3 Center of Medical and Bio-allied Health Sciences Research, Ajman University, Ajman P.O. Box 346, United Arab Emirates; k.aidinis@ajman.ac.ae; 4 Department of Electrical and Computer Engineering, Ajman University, Ajman P.O. Box 346, United Arab Emirates

**Keywords:** paper-based electronics, paper-based field-effect transistors (PFETs), paper-based sensors, paper-based biosensors

## Abstract

Paper-based electronics have emerged as a sustainable, low-cost, and flexible alternative to traditional substrates for electronics, particularly for disposable and wearable applications. This review outlines recent developments in paper-based devices, focusing on sensors and paper-based field-effect transistors (PFETs). Key fabrication techniques such as laser-induced graphene, inkjet printing, and screen printing have enabled the creation of highly sensitive and selective devices on various paper substrates. Material innovations, especially the integration of graphene, carbon-based materials, conductive polymers, and other novel micro- and nano-enabled materials, have significantly enhanced device performance. This review discusses modern applications of paper-based electronics, with a particular emphasis on biosensors, electrochemical and physical sensors, and PFETs designed for flexibility, low power, and high sensitivity. Advances in PFET architectures have further enabled the development of logic gates and memory systems on paper, highlighting the potential for fully integrated circuits. Despite challenges in durability and performance consistency, the field is rapidly evolving, driven by the demand for green electronics and the need for decentralized, point-of-care diagnostic tools. This paper also identifies detection strategies used in paper-based sensors, reviews limitations in the current fabrication methods, and outlines opportunities for the scalable production of multifunctional paper-based systems. This review addresses a critical gap in the literature by linking device-level innovation with real-world sensor applications on paper substrates.

## 1. Introduction

Nowadays, the semiconductor and electronic device landscape is evolving around new needs and challenges. The global semiconductor shortage has changed the way devices are designed and manufactured [1,2,3], distributed [4], and bought from end users, forming new consumer patterns based on mass demand [5,6]. More specifically, various technological sub-sectors have been affected by the phenomenon, such as [3] fabless industries that design devices and produce IPs, foundry-level enterprises that produce en masse, and outsourced semiconductor assembly and test (OSAT) merchant vendors. It is interesting that a major contributor to this global crisis was the sudden increase in demand for mid-range electronics, while all manufacturers had shifted their production weight towards cutting-edge technology components [1]. Additionally, emblematic initiatives such as the European Green Deal [7] pave the way for a new, more sustainable way of producing electronics in general [8,9]. This can be achieved (a) by using processes that have low material waste, low byproduct production, and high throughput/yield with low operation costs [10,11], and (b) by using more environmentally friendly materials [12].

Regarding innovative processes, we can identify various printing techniques [13] that can be utilized for electronics manufacturing, either additive or subtractive, such as inkjet [14,15], screen printing [16,17], aerosol jet [18,19], electrohydrodynamic jet [20,21], and various roll-to-roll techniques (with gravure [22,23], flexo [24], and offset [25] being the main examples). The integration of high-throughput manufacturing processes, such as printing, into electronics fabrication offers significant advantages. Due to the additive nature of certain printing techniques, material waste can be minimized. Moreover, fabrication speeds can increase by orders of magnitude, as these processes are capable of processing substrates at rates measured in square meters per second. In addition, the use of innovative, printing-compatible materials enables the further enhancement of device performance. Material-wise, a material with a greater contribution to both environmental impact and cost should be used as the device substrate, given the fact that this is always the bulkier component and the limiting factor for the process. Ideally, for the mass production of low-cost electronics, such as sensor tags, single-use test strips, and so on, the substrate should be partially recyclable, readily available, non-toxic to humans and the environment, and easy to handle. One such candidate is paper and its various modified versions.

**Figure 1:**
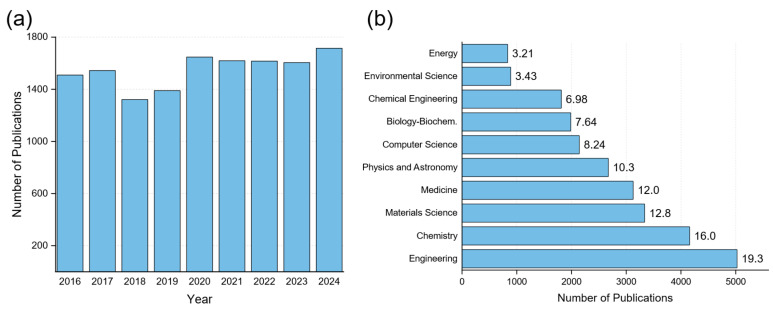
Publications per year involving paper-based-devices-related keywords (**a**); percentage breakdown by major scientific sector (**b**).

Paper, as a substrate for the development of various devices and sensors, is the main subject of this review. Paper stands out due to its biodegradability, low cost, widespread availability, and compatibility with various low-temperature fabrication techniques such as inkjet and screen printing. Unlike polymeric substrates, paper enables capillary action and microfluidic channeling, making it inherently suitable for both sensing and fluid handling in integrated systems. These properties position paper as a uniquely capable platform for developing disposable, point-of-care, and wearable electronics. There are some very comprehensive reviews on paper-based electronics, which investigate this topic from various angles: broader approaches [26], such as human-centric applications such as wearables, skin-friendly, or biological devices [27,28,29,30], application-oriented reviews for sensors [31,32,33,34], devices [35,36], and microfluidics [37,38,39,40], and eco-aware and green devices [41] are some examples. Nevertheless, the field is rapidly evolving, and new applications are emerging constantly. Therefore, this review aims to highlight recent advances in the field after briefly discussing some necessary introductory elements, such as material utilization and the most common patterning techniques. Queries from the literature were performed using Scopus, a well-recognized tool for its efficiency and validity [42,43]. With an initial query for a year range of 2016 to 2024, and keyword searches for “paper” or “cellulose” and the terms “sensor”, “biosensor”, “electrochemical”, “device”, “FET”, “TFT”, “capacitor”, “diode”, “memory”, and “transistor”, 13,966 documents were retrieved (Figure 1a). An interesting observation is that while most publications were correlated with engineering sciences, various other sectors were referenced, such as medicine, chemistry, energy, and so on (Figure 1b). This can lead to the conclusion that such innovative devices, although derived from engineering backgrounds, provide solutions for various problems in a wide range of applications.

**Figure 2:**
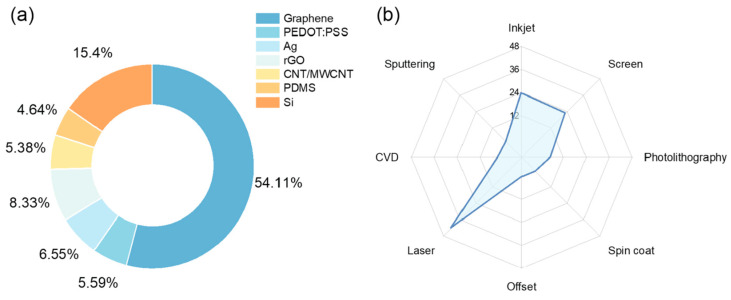
Common materials utilized for developing electronics with paper substrate (**a**); percentage of fabrication techniques for developing electronics on paper (**b**). Both percentages are extracted from the articles investigated in this review.

By examining the basic materials utilized in developing several types of devices on paper, we can easily detect the importance of graphene and its strong coupling with the paper substrate. More specifically, 54.11% of the research works surveyed included graphene in some form, while 8.33% used reduced graphene oxide (rGO) [44]. PEDOT/PSS and carbon nanotube-based materials such as single and multi-walled carbon nanotubes share similar percentages, while silicon and PDMS have been vastly referenced, each for a different role; typically, PDMS can be used as a passivating material or as a patterned gasket–microfluidic channel, while silicon has been mostly referenced for being used at various steps in manufacturing (micromachining–micropatterning and so on) (Figure 2a).

Laser (with laser-induced graphene) has a major contribution as a technology for developing paper-based electronics. Following that, printing techniques such as inkjet and screen printing have been widely used. Other traditional microelectronic fabrication processes have been utilized as well, at various stages of a device development. Figure 2b presents a percentage of referenced technologies in the works under investigation. This interesting observation on the one hand indicates that paper substrate is compatible with a variety of processing technologies, and on the other hand the diversity of devices that can be fabricated onto it, depending on the application requirements.

While several reviews have examined paper-based electronics, they often focus broadly on material aspects [26], human-centric applications [27,28,29,30], or specific sensor types [31,32,33,34]. However, there is limited comprehensive evaluation of paper-based field-effect transistors (PFETs) and their dual role in both sensing and device-level computation. This review aims to fill that gap in the following ways:

- Analyzing the integration of PFETs in modern paper-based systems;
- Categorizing PFETs by application domain (e.g., biosensing, logic gates, physical sensing);
- Evaluating detection techniques and critical performance metrics.

By highlighting key trends, fabrication approaches, and functional performance parameters, this review provides a structured and up-to-date overview of recent developments in the field and identifies future opportunities and challenges for scalable, multifunctional paper-based electronic systems.

**Figure 3:**
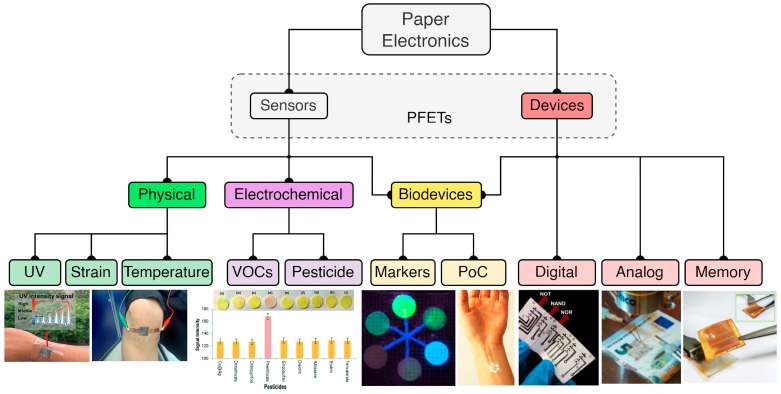
An overview of paper electronics applications and examples: from wearable physical sensors and biosensors to biodevices (UV, humidity, temperature, and more), electrochemical detectors for VOCs, and devices and FETs for logic gates, analog FET arrays, and memory arrays, the field is full of innovative solutions. Images reproduced with permission from [45] copyright 2022, American Chemical Society from [46] copyright 2020, Royal Society of Chemistry from [46] copyright 2020, Elsevier from [47] copyright 2021, Wiley from [48] copyright 2019, Wiley from [49] copyright 2016, Royal Society of Chemistry.

More specifically, this work aims to present, organize, and categorize recent works found in the literature from the most widely covered sectors involving paper-based electronics. We have divided the paper into discrete sections, as presented in Figure 3. The basic sectors are sensors and devices. Applications of PFETs include both sensors and devices; thus, they are considered as a broader category in this work. Sensors are devices that detect and respond to physical stimuli by converting the measured parameter into an electrical signal that can be interpreted, processed, or recorded. On the other hand, devices (with a typical example being a transistor) are electronic components that can perform signal processing, computation, or control, such as amplification, switching, or signal retention. These main categories are followed by a more application-specific analysis, where sensors can be used either for sensing physical quantities such as UV radiation, strain, and temperature or as electrochemical sensors that can be used for detecting volatile organic compounds (VOCs), pesticides, and more. Active devices based on paper substrate can be digital electronics arrays (logic gates such as NOT, NAND, etc.) or analog electronics, with various transistor arrays being developed recently on paper and memory systems. Biodevices represent an important sector where various groups have proposed systems based on either sensors or devices; therefore, biodevices are categorized under both sensors and devices.

## 2. Paper-Based Sensors

Paper-based sensors not only use paper as the structural material but also, in many cases, utilize the physical and chemical properties of paper for detecting and quantifying various target quantities or analytes. These sensors are inexpensive, easy to use, and can be produced in large quantities. They are typically composed of a combination of different paper substrates, conductive materials such as metallic nanoparticles and graphene-based materials, and functional materials such as enzymes, antibodies, functional polymers, etc. The response (either optical or electrical) to a specific stimulus is then measured and the parameter that is correlated with the stimulus is quantified. In the following sections, we have divided the greater sensor groups based on the target parameter of the substance; all the mentioned works are developed on different types of paper substrate, and there are numerous strategies for both patterning and sensor fabrication, and for sensing and the underlying mechanism involved. Despite this diversity, the central feature that consistently stands out is the versatility of paper as a sensor platform. Its low cost, biodegradability, and adaptability enable innovative sensing solutions that range from simple visual assays to complex, multi-parameter electrochemical systems.

### 2.1. Pesticide—Heavy Metals Detection

**Figure 4:**
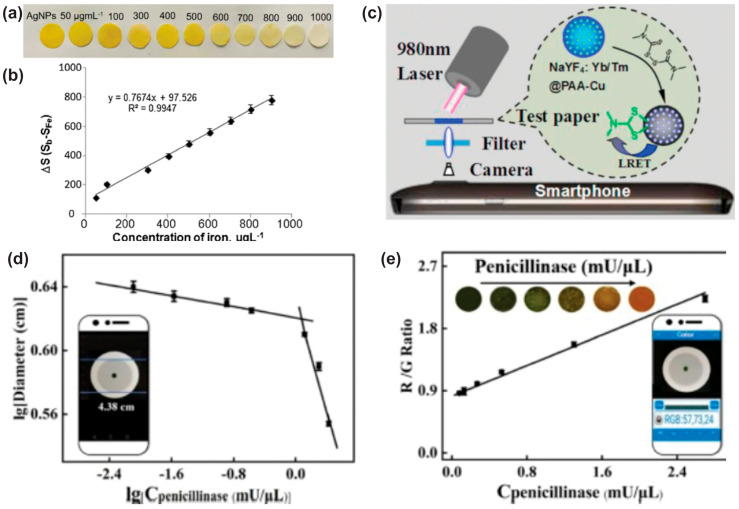
(**a**,**b**) Optical detection of iron concentration in water and blood plasma (reproduced with permission from [52] copyright 2020, Springer Nature); (**c**) pesticide detection with smartphone and paper device (reproduced with permission from [53], copyright 2016, Elsevier); (**d**,**e**) microbial detection via camera (reproduced with permission from [54], copyright 2021, Elsevier).

While more traditional, electrical output sensors are used for numerous applications with high accuracy and performance, optical (colorimetric, chromatographic, and fluorescent) sensor systems are equally attractive mainly for their facile readout which can be either direct human interpretation of the results (e.g., a color change indicates the presence of an analyte), or more detailed measurements by means of a smartphone camera, given that nowadays portable and smartphone cameras have unprecedented resolution although even lower-end, older cameras have been demonstrated to be capable of working for green analysis on-site [50]. In fact, the combination of smartphones and sensors is a mass scale trend with a handful of modern applications. Modern examples of this type of system are presented in Figure 4, where paper-based systems are used in conjunction with a smartphone, which is utilized as the detector of the quantity under measurement. More specifically, sulfate quantification [51], determination of Fe^3+^ in water and blood plasma using cetyltrimethylammonium bromide modified AgNPs-impregnated paper [52] (Figure 4a,b), pesticide thiram detection [53] (Figure 4c), and penicillinase detection using a customized bromothymol blue–cetyltrimethylammonium bromide–alginate complex filter paper for color indication [54] (Figure 4d,e) are some indicative examples. In an extensively discussed manner, Biswas et al. [55] exhibited a set of advantages of such systems, coined “S.M.A.R.T”, for their smartphone integration, minimal sample volume, affordability, rapidness, and testing in the field. For a system that can detect hemoglobin concentrations on-site in approximately 5 min, the cost is about 0,02 USD/test.

### 2.2. Physical Sensors

**Figure 5:**
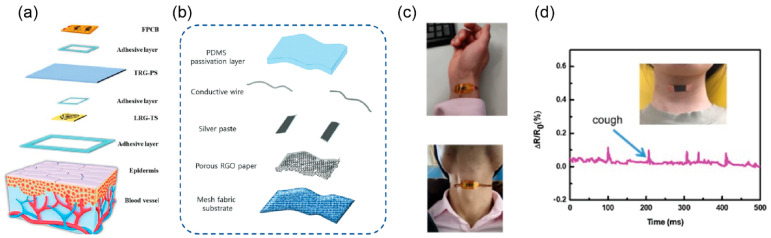
(**a**) A wearable, washable, and breathable pressure–temperature skin sensor (reproduced with permission from [56], copyright 2021, Elsevier); (**b**) UVA flexible, wearable patch (reproduced with permission from [57], copyright 2022, Wiley); (**c**) pressure sensor from graphene porous paper installed on human body (reproduced with permission from [59], copyright 2022, Springer Nature); (**d**) strain sensor on a mulberry paper substrate (reproduced with permission from [61], copyright 2020, Elsevier).

The most common physical properties that attract research interest are temperature, relative humidity, UV radiation, strain, and pressure (Table 1). By coupling technologies for disposable sensor fabrication with modern materials, a wide range of applications can be covered solely by paper-based electronics. Indicative applications of physical sensors are presented in Figure 5; it can be observed that the paper substrate can act as a carrier for a variety of wearable sensors for the facile detection of critical physical parameters. Zhang et al. [56] demonstrated a washable and breathable laser-reduced graphene cellulose temperature sensor, and a thermally reduced graphene-cellulose pressure sensor integrated with flexible electronics for data transmission from human skin (Figure 5a). An ultraviolet radiation patch sensor based on modified paper with reduced graphene oxide was presented in [57] (Figure 5b), with results comparable to commercial UVA sensors and an advantage over substrate bendability. Wastepaper was graphene-coated and used for measuring pressure and strain in [58]. The wastepaper was converted into a pulp and underwent oxidation, reduction, and dispersion in an ethanol solution of graphene, followed by oven-drying, forming a porous conductive aerogel. Similarly, porous graphene paper was prepared with PMMA microspheres as a template [59] with a fast response time (<60 ms) in detecting on-body strain (Figure 5c). An alternative route in fabricating microporous reduced graphene oxide paper was proposed by the same authors, where a self-evaporation method was utilized was recently presented with comparable results [60]. Custom mulberry paper was coated by the Meyer-rod coating method with graphene solution for the development of a wearable strain sensor in [61] (Figure 5d). This work also investigated the importance of coating thickness and presented proof of detecting throat and hand movement, as with similar research presented herein.

### 2.3. Humidity Sensors

**Figure 6:**
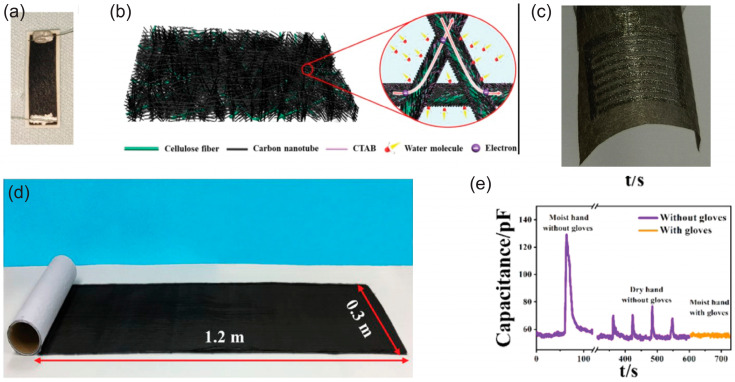
(**a**) Example of a paper-based humidity sensor produced with LIG (reproduced with permission from [65], copyright 2022, Wiley); (**b**) humidity sensor based on a TEMPO-oxidized cellulose fibers/carbon nanotubes fiber network (reproduced with permission from [67], copyright 2021, Elsevier); (**c**) self-assembled graphene-oxide sheets form a layer for humidity sensing as well (reproduced with permission from [68], copyright 2019, Elsevier); cellulose nanofiber/CNT nanoporous sensitive coating for paper can be used to produced rolls of humidity-sensitive paper (**d**) [69]; wood-derived cellulose paper modified for sensing skin moisture typical response (**e**) [72].

Paper-based humidity sensors are commonly used as resistive-output or capacitive-output devices, exploiting cellulose’s high humidity absorption and, consecutively, modulation of its permittivity and electrical resistance. Recently, devices as simple as graphite-on-paper (pencil drawn and cut patterns) have been presented with overall satisfactory results and an exceptionally low cost of production [63]. In Figure 6, we present modern approaches for developing humidity sensors based on paper substrate with the addition or patterning of active materials over it. Irradiating paper to induce graphene by laser (LIG, laser-induced graphene) has been used in [65] (Figure 6a) to create sensors with high sensitivity (up to 1.3 × 10^−3^%RH^−1^). Alternative approaches include the development of sensors with a custom, activated cellulose substrate; a 2,2,6,6-tetramethylpiperidine-1-oxyl (TEMPO)-oxidized cellulose fibers/carbon nanotubes (TOCFs/CNTs) conformal fiber network humidity sensor was presented in [67] (Figure 6b) with outstanding linear response, biodegradability, and a wide sensing range under bending. This approach is unique to the fabrication technique, because it uses an electrical field for the assembly of the sensing layer on charged cellulose. A dual cellulose nanofiber/CNT nanoporous sensitive layer was proposed in [69] (Figure 6c) for both fast exchange of water molecules between the environment and the sensor, and for improved adhesion onto the substrate, effectively creating sensor rolls, which can be cut out in varied sizes. Guan et al. [71] used glycidyl trimethyl ammonium chloride (EPTAC) and screen-printed silver electrodes, claiming that increased hydrophilicity increased the sensor response. The authors also demonstrated a skin-breath (distance up to 14 mm) and a human breathing detection application. The filtration of rGO/PANI with paper can be used to deposit the desired sensing material on the substrate [62,64]. Carbon-based materials are used for their simple humidity-sensing mechanism: hydrophilic groups such as hydroxyl (–OH) and epoxy (=O) naturally exist on these materials’ surface, and water molecules bond with them and remove electrons, causing the electrical conductivity of the material to drop [68] (Figure 6d). Wang et al. [72] in 2020 exhibited a smart and green way for developing humidity sensors, using conductive wood-derived cellulose nano-paper. These devices have been demonstrated to be capable of detecting both breathing, and skin moisture desorption at distances of up to 2 cm (Figure 6e). Additionally, porous paper can be used as a substrate; it can be derived from standard copy paper following a straightforward process that involves mild etching-like processing with HCl to remove the CaCO_3_ filler, thus assisting both in printing the active material and in humidity absorption. An example is presented by Zhang et al. [73], in which the process is followed, and highly repeatable devices have been fabricated. Another interesting application field in humidity sensing is human respiration analysis, both in terms of physiochemical contents, and in terms of physiological features (rate, rhythm, anomalies, etc.) [86]; paper-based sensors can be used in this application field with different mounting and sensing strategies [73,87]. An interesting approach in biocompatible humidity sensing was the coating of edible rice paper with a triboelectric nanogenerator, fabricating an energy-autonomous humidity sensor in full relative humidity range with a fast response and recovery time (5/16 s) [76].

### 2.4. Electrochemical Sensors—Biosensors

Paper-based electrochemical sensors are analytical systems that utilize paper substrates combined with electrochemical detection methods to measure chemical or biological analytes. They typically consist of conductive electrodes printed or deposited on paper, enabling electrochemical reactions such as oxidation or reduction, which generate measurable electrical signals (e.g., current, voltage, or impedance).

Various air quality factors have been shown to be able to be measured with a small, facile wearable system based on dip-coated paper in conductive carbon black/exfoliated graphene [62]. This work discussed the successful detection of relative humidity, volatile organic compounds (VOCs) (methanol, ethanol, toluene, dichloromethane, acetone, and petroleum ether). Butanol was successfully detected as well [88]. Other toxic gases such as H_2_S at concentrations as low as 1 ppm at room temperature were detected with an inkjet-printed CuAc (copper acetate) sensing film on multi-coated paper substrate with polymerized carboxylated styrene butadiene acrylonitrile copolymer and polystyrene [89]. This film provides a dual-sensing mechanism (electrical and optical—colorimetric). Following the need to detect toxic residues with low cost, disposable devices, Jemmeli et al. [90] have recently presented a reagent-free detection system for sensing bisphenol A (BPA), an endocrine disruptor, in water. The whole sensor was printed onto filter paper with carbon black and Ag/AgCl electrodes by screen printing and BPA was successfully detected in sampled river water via cyclic voltammetry.

**Figure 7:**
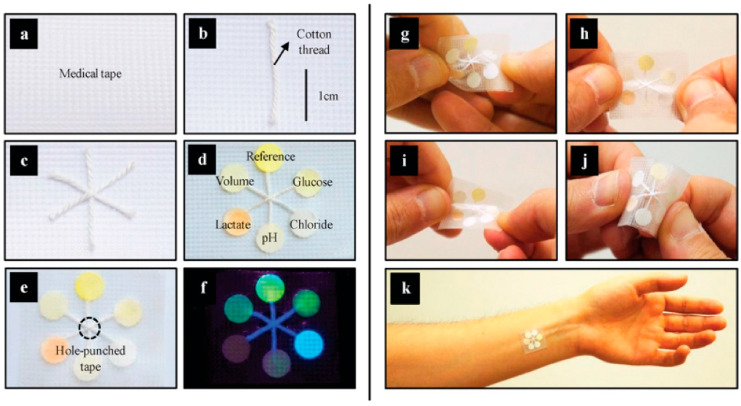
Complete fabrication process for a set of fluorescent probes for biomarker detection in sweat. (**a**) A strip of medical tape, (**b**) a 2 cm cotton thread, (**c**) a cross-shaped arrangement of three cotton threads affixed to the tape, (**d**) fluorometric assays printed on paper, (**e**) a piece of medical tape with a punched hole placed on top, (**f**) the sweat patch viewed under UV light, (**g**) the patch being twisted, (**h**) the patch being stretched, (**i**) simultaneous twisting and stretching of the patch, (**j**) bending of the sweat patch, and (**k**) the sweat patch applied to forearm. reproduced from [93] copyright 2020, Elsevier.

**Figure 8:**
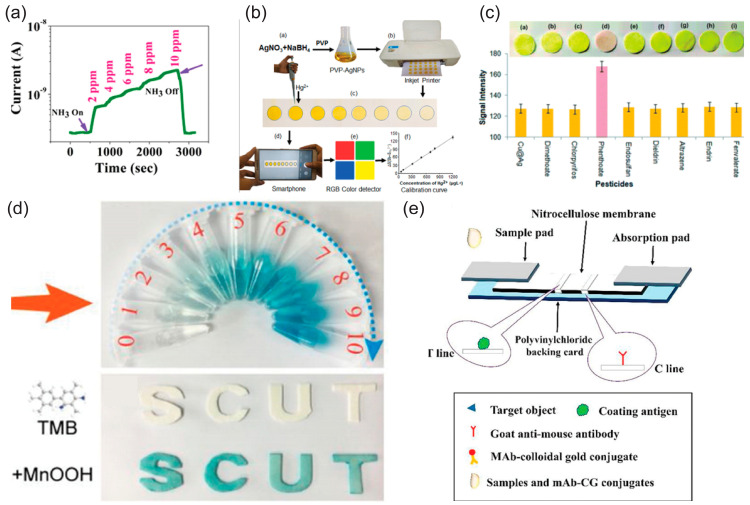
(**a**) NH_3_ gas sensing with a low detection limit (reproduced with permission from [89], copyright 2019, Springer Nature); (**b**) optical detection via smartphone of Hg^2^⁺ (reproduced with permission from [94], copyright 2021, Elsevier); (**c**) selective optical response to phenthoate (reproduced with permission from [46], copyright 2020, Royal Society of Chemistry); (**d**) MnOOH-TMB reaction on custom paper shapes (reproduced with permission from [96], copyright 2019, Elsevier); (**e**) test strip for detection of toltrazuril (Tol) (reproduced with permission from [100], copyright 2019, Elsevier).

A wearable biocompatible system based on paper/thermally reduced graphene oxide/nylon was developed, exhibiting a high performance in measuring electrocardiography, electroencephalography, and electromyography signals, as well as human motion [91]. Skin perspiration (and sweat loss) can also be monitored with a CNT/paper-based patch, as recently shown in a communication article [92]. In a material-weighted approach, Ardalan et al. [93] (Figure 7) presented a patch with discrete, fluorescent probes for indication of biomarkers such as glucose, lactate, chloride, pH, and volume. The readout takes place in a contactless manner, using a smartphone application. In Figure 8, we have gathered a set of electrochemical paper-based sensors which are fabricated using various techniques, such as printing, mechanical patterning and cutting, overcoating, etc. Additionally, these systems exhibit the variety of sensing mechanisms presented in the modern literature of electrochemical paper-based sensors.

Toxic residues such as organophosphorus pesticides can be fluorescently identified using a paper-based device with 100% accuracy [94]. NH_3_ with a very low concentration limit and high selectivity has been developed on paper substrate with a grown active layer of perovskite halide (MAPI) [95] (Figure 8a); the sensor works without the need for a heater for additional sensing assistance, and provides an order-of-magnitude change in the electrical current response to 10 ppm NH_3_.

Graphene paper can be used as a carrier substrate for Bi/Nafion electrodes, for the detection of Cd^2+^ and Pb^2+^ in water, for quality monitoring using disposable means [96]. Heavy metal ions have also been detected with ZnO-NPs, rGO, and ethylenediaminetetraacetic acid (EDTA) [97]; optical detection of Hg^2+^ in environmental water samples with AgNP printed on Whatman paper sensor has been demonstrated and cross validated with inductively coupled plasma–atomic emission spectroscopy (ICP-AES) results. The presented sensing mechanism was robustly proven with (UV–Vis) spectrophotometry, TEM, XPS, DLS, and basic chemical assays [98]; a typical end-to-end process for the fabrication and field deployment of the mentioned sensor system is presented in Figure 8b. In a similar manner, aggregation-induced emission was exploited to sense Cd^2+^ in water; the paper was engineered to transmit both the background (reference) color and the detection color with a detection limit of 33.3 nM [99]. Phenthoate pesticides for food safety can be detected using paper sensors with AgNPs/CuNPs, owing to the high affinity of phenthoate to interact with the specific material stack, which in turn results in a color change on the sensor strip. This work presented a well-designed device with explementary selectivity across other pesticides [46] (Figure 8c). An example of the flexibility that paper substrate provides as a sensor can be seen in Figure 8d, from [100], where sensor strips have been mechanically patterned as letters. Typically, enhancement of the optical results can be achieved using artificial intelligence [101]. Structures with multiple materials can be fabricated in a compact, strip-form factor with the addition of selective antibodies (Figure 8e). A table which includes the most important parameters for this family of sensors gathered together for comparison is presented below (Table 2).

Graphene and rGO-based sensors on paper substrate have a wide area of applications, with biological and analytical devices playing a key role [44]. A paper-based analytical device for creatinine sensing with a high degree of specificity against other common interferences (urea, uric acid, glucose, and ascorbic acid) has been demonstrated with a copper oxide–ionic liquid/reduced graphene oxide composite [105]. The study evaluates rGO reduction cycles and concludes on the importance of the large-scale manufacturing of such arrays with printing. A screen-printed cholesterol biosensor was recently presented [106] with a graphene oxide–polyaniline composite. A graphene oxide-based screen-printed electrode for DNA sensing can be used to effectively address the drawbacks of traditional RT-PCR [107], while the appropriate chemical modification or activation of screen-printed electrodes can be used to make them electroactive against particular biomolecule levels in people, such as phenylalanine (PHE), important in patients suffering with phenylketonuria (PKU) [108]. Paper-based biosensors can also be used to detect Human Papilloma Virus [109], traces of Furazolidone (an antibiotic responsible for mutations and carcinogenesis) [110], and water-borne bacteria [111,112]. Additionally, antibiotic sensing (Tetracycline) using a paper-based device with an test-on-site manner has been recently developed [113].

#### 2.4.1. Glucose Sensors

Glucose monitoring is vital for assisting various endocrine metabolic diseases, with diabetes being one of the most serious. Paper-based PoC (point-of-care) devices have played a significant role in this task [114]: Yang et al. [115] demonstrated a glucose paper-based sensor with graphene wall and nano-Cu_2_O modified paper. Graphene walls were tethered onto the substrate by chemical vapor deposition, and Cu_2_O was grown onto the graphene walls by thermal decomposition. This advanced fabrication strategy allowed for a detection limit of 0.21 μM and a response time of less than 4 s. Further modifications in graphene-based paper with ultrafine PtCo alloy nanoparticles improved selectivity [116]. Graphene paper with CuO/Cu(OH)_2_ nanostructures prepared with thermal/laser modification has been used in the amperometric detection of glucose with a linear behavior between 50 μM and 10 mM [117]. Other non-enzymatic approaches using carbon-based materials on paper substrate have recently been presented [118,119,120,121,122,123,124,125]. A Prussian blue–reduced graphene oxide–tetraethylene pentamine (rGO-TEPA/PB) screen-printed electrode on top of a microfluidic channel fabricated with photolithography processing with hydrophilic modified zones has been demonstrated to effectively work linearly in the range of 0.1–25 mM [126], while the combination of Prussian blue–graphenic materials has demonstrated a high sensitivity of 1539.53 μA mM^−1^ cm^−2^ for fabricating glucose-sensing electrodes [127]. A supporting material decorated with Cu-Pd nanoparticles, such as reduced graphene oxide, was found to assist in the detection of glucose oxidase with a detection limit of 0.29 μM, with colorimetric output on paper-based strips [128]; similarly, nickel and nitrogen-doped graphene nanotubes decorated with Pt nanoparticles have been used to detect glucose with colorimetry as well [129]. Graphene oxide has also been used with chitosan [130,131], ionic liquids (with Prussian blue and MXene) [132], or other conductive materials [133]. Alternative readout systems can include smartphones, enabling the at-home monitoring of glucose with a paper-based device [134]. A paper-based electrochemical sensor modified with nickel-based metal–organic frameworks (Ni-MOFs) for enzyme-free glucose detection was fabricated in [135]; the device was developed onto a paper substrate with simple processing steps (namely screen printing and drop casting), and a hydrogen peroxide sensor was incorporated as well, underlining the possibility of multi-parameter sensor development on paper substrates. In a similar manner, a multi-parameter biosensing system was presented recently by Cheng et al. [136], capable of sensing lactate, uric acid, magnesium ions, pH, and glucose, all with colorimetry. A comparative analysis of critical parameters on glucose sensors based on paper is presented in Table 3.

#### 2.4.2. Cancer Sensors

Cancer detection, both for early detection and for treatment monitoring, is a fundamental problem, especially when considering resource-constrained frameworks, where it is vital to have cost-effective point-of-care devices. By adopting paper as a building block for such tests and sensors, disposable and fully compatible systems can be developed for various cancer cells’ and markers’ detection. There have been various demonstrations based on different working principles, with some indicative recent advances being the following: Zhou et al. [140] presented a graphene oxide–Au nanocomposite nitrocellulose paper-based assay for breast cancer detection, using the photo-thermal effect. A similar material stack (rGO/Thi/AuNPs) was used in [141], but it exploited the current response of thionine, which was found to be proportional to a cancer antigen. An integrated wireless point-of-care system which was capable of detecting neuro-specific enolase (NSE), significant to the prognosis and monitoring of small cell lung cancer, was presented recently [142]; the device was based on differential pulse voltammetry, thionine and gold nanoparticles were used as sensing materials (a combination seen in other works as well [143]), and the biodevice results were stored in an EEPROM memory so that they could be displayed by a smartphone application.

An enzyme-free analytical device, based on gold nanowires and graphene oxide, with CuS nanoparticle/graphene oxide signal labels was fabricated by screen printing, for the detection of α-fetoprotein [144]. Another innovative approach is the use of aptameric graphene oxide sensors on paper, for the fluorescent detection of three different cancer cell lines, namely MCF-7, HL-60, and K562, with the use of a single light source [145]. Such a system is considered highly promising, for its accessibility and ease of use. An electrochemical sensor on paper for pancreatic cancer biomarker detection with gold nanoparticles, carbon-Ag/ACl electrodes, and SU8 prepatterned paper was recently demonstrated by Prasad et al. [146] with a limit detection of 10 pgmL^−1^. The paper uses graphene oxide as an overcoating layer for facile antibody–electrode linking. Ovarian cancer, due to its severity, is a major problem to probe and monitor; a bio-microfluidic system on paper, based on Ag/rGO, was developed for the detection of the cancer tumor protein CA 125 by immobilizing anti-CA 125 antibodies with the assistance of cysteamine-coated gold nanoparticles on the paper-based electrodes [147]. An alternative route for the bio-safe and cost-effective patterning of electrodes is the use of poly(3,4-ethylenedioxythiophene)/poly(4-styrenesulfonate) (PEDOT/PSS) with various modifications and the immobilization of antibodies for cancer detection; PEDOT/PSS/rGO-coated Whatman paper after PEDOT/PSS standard conductivity enhancement with EG and immobilization of monoclonal carcinoembryonic antibodies was tested for repeatability in fabrication with good results (a relative standard deviation of 7.6% in the current response for five devices) and selectivity between different analytes in serum samples [148]. Similarly, the detection of carcinoembryonic antigen with a PEDOT/PSS/graphene electrode based on a paper substrate was investigated with electrochemical impedance spectroscopy in [149]. It has been shown that a similar detection performance can be achieved with decorated PEDOT/PSS with nanostructured iron oxide (nFe_2_O_3_@PEDOT/PSS) on Whatman paper [150]. Multi-walled carbon nanotubes (MWCTNs), bioactivated with an appropriate antibody for the detection of prostate specific antigen and electrical wires stabilized with silver paste, were fabricated by Ji et al. [151]; the sensor exhibited a maximum response of 150% change in electrical resistance in the presence of PSA. Finally, a novel three-dimensional plasmonic hexaplex nanostructure was used, in conjunction with a surface-enhanced Raman scattering of human saliva samples, to detect possible malignant patients [152].

### 2.5. μPADs

Paper-based microfluidic analytical devices (μPADs), in a more integrated manner than the separate devices presented in the previous paragraphs, are complex projects with a variety of parameters to be considered. The research attraction for this subject is so high that it requires a separate, dedicated review to be covered thoroughly; therefore, it will be considered out of the scope of the present work. The authors have identified some very recent articles that cover the subject in detail: starting from Carrell et al. [153] who discussed the evolution of portable, low-cost paper-based tests from simple lateral flow assay tests to more compact systems, to a technical analysis of materials and methods for fabrication [154,155], and application-specific breakdowns [156,157]. The reader is encouraged to follow the references for in-depth discussion of the subject. Nevertheless, some recent advances in the field shall be presented herein for a general understanding. Chaiyo et al. [158] presented a highly sensitive and selective device for the determination of Pb(II), Cd(II) and Cu(II). The device combines both electrochemical and colorimetric responses in an innovative manner. Hydrophobic barriers have been formed on chromatographic paper using chemical vapor deposition, for glucose assays, immunoassays, and heavy metal detection [159]; these devices provide a cost-effective platform for portable testing with a low limit of detection. Mercan et al. [160] showed the utilization of machine-learning platform for the accurate glucose determination with a μPAD. A microfluidic paper-based device for cortisol detection in human sweat was recently presented [161]; this device incorporates the active layer, screen-printed electrodes, a microfluidic channel, and a wireless front-end for data communication with a remote application, all developed onto a paper substrate.

## 3. Paper-Based Field-Effect Transistors (PFETs)

A wide variety of flexible electronic applications often rely on FETs that are typically produced on polymeric substrates [162,163,164,165,166,167]. However, due to the considerable environmental impact of the plastic waste, in the last decade, researchers have explored the use of paper as an alternative substrate [7,8,9]. Despite their more complex manufacturing process compared to passive electronic components, several research groups have developed paper-based transistor devices using a variety of semiconducting, insulating, and conductive materials and fabrication processes [26,35,168,169]. The selection of these materials and processes is determined by numerous factors such as device performance, application field, stability, repeatability, material waste reduction, etc. Large-scale fabrication of these devices typically involves the use of a common deposition and patterning technique, or a combination of them. As the fabrication process of PFETs is becoming more accessible and their performance is improving, they are becoming viable for a wide range of potential applications. These devices have unique properties that make them attractive for various fields, such as biosensors [170,171], wearable electronics [172,173], smart packaging [174,175], medical diagnosis [175,176], etc. Despite the promising outlook, the development of PFETs is still in its initial stages, and ongoing research is exploring new applications.

**Figure 9:**
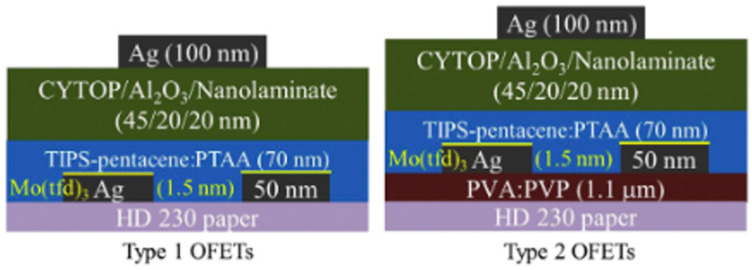
Top-gate OFETs on bare and on PVA/PVP-coated commercial HD 230 paper substrate (reproduced with permission from [180], copyright 2017, Elsevier).

PFETs’ basic components usually include a dielectric layer, a semiconducting channel, source–drain electrodes, and a gate electrode. The channel is typically one or more thin layers of a conducting or semiconducting material and is located between the source and drain electrodes. The third terminal of the transistor is the gate which is separated from the channel by a thin dielectric layer. The gate controls the current flow between the source and drain by modulating the electric field across the dielectric. P-type and n-type materials are typically either inherently semiconducting or require doping to exhibit the desired behavior. Several methods have already been introduced for creating n-type and p-type paper-based transistors, including chemical doping, electrochemical doping, vapor deposition, etc. [168,177,178,179]. When an electric charge is applied to the gate electrode (positive or negative) of a PFET, it can attract electrons or holes in the channel depending on the semiconductor type (n-type or p-type) and the transistor mode (enhancement or depletion). Several types of PFETs, including top gate, bottom gate, and floating gate, are manufactured based on their intended use and specific application requirements. Figure 9 depicts the schematic of two top-gate organic field-effect transistors fabricated on commercial paper.

There are several parameters that indicate the functionality of a PFET that include the following: the transfer characteristics (I_DS_/V_DS_ and I_DS_/V_GS_) which are used to evaluate the overall performance and the transistors’ stability; the charge carrier mobility μ which is an important parameter to characterize the speed of the charge transport; the I_ON_/V_OFF_ ratio which characterizes the FET’s ability to turn the active channel on and off; the threshold voltage which is a measure of the FET’s switching performance and is an important factor in determining its suitability for various applications; the device geometry ratio W/L which determines the dimensions of the channel and the gate region; ion sensitivity in the case of ion-sensitive applications, etc. Compared to conventional transistors, characterizing a PFET transistor is a non-trivial process due to the complex parameters and conditions that significantly influence its functionality. The fabrication process, doping concentration, gate oxide thickness, channel length, and temperature are also factors that affect the functionality of PFET transistors. The fabrication process can influence the transistor’s threshold voltage and mobility, while the doping concentration and the semiconductor type affect its carrier concentration and mobility. Gate oxide thickness is critical for controlling the threshold voltage and subthreshold swing, while channel length impacts the output resistance and saturation current. The choice of dielectric material also plays a key role in transistor performance, as it influences the gate capacitance and the interface trap density. The dielectric constant, breakdown voltage, and thermal stability of the dielectric material must be considered to ensure reliable transistor operation. Moreover, operating temperature significantly affects the performance of the transistor, particularly in terms of carrier mobility, threshold voltage, and leakage current. Therefore, to effectively characterize PFET transistors, one must consider all these variables and their interdependencies, as well as the impact of external factors on device reliability. It is essential to conduct a comprehensive and systematic analysis of transistor behavior to ensure reliable and consistent device operation. As a result of the growing demand for unique properties such as biocompatibility and eco-friendliness, several types of PFETs have been developed using novel fabrication methods, new substrates, and semiconducting materials. Several works have been carried out in this field, resulting in the production of low-voltage PFETs with acceptable performance characteristics. Although the attainment of performance levels comparable to conventional FETs is challenging due to limitations such as stability, sensitivity, geometry power consumption, charge carrier mobility, operating voltage, etc., research on PFETs has yielded interesting results, positioning them as promising candidates for a wide range of applications.

PFETs can be classified into various categories based on their fabrication process, device architecture, and mechanism of current modulation. The fabrication process determines the method used to deposit the conductive and semiconductive materials on the paper substrates such as solution-processed transistors, direct-writing transistors, roll-to-roll printed transistors, etc. Device architecture refers to the design of the transistor and can be classified as single-layer, double-layer, multilayer, vertical transistors, etc. Finally, PFETs could be categorized based on the technique materials used to modulate the current flow to organic thin-film transistors (OTFTs), paper-based electrolyte-gated transistors, graphene and carbon nanotube-based transistors, etc. Indeed, several works [26,35,168,169] have already presented systematic analyses and reviews based on these types of categorizations, especially focused on paper materials and deposition techniques. Therefore, in this work, we decided to categorize PFETs according to their application field. Categorizing PFETs based on their application provides a targeted and focused analysis of the transistor’s behavior, enabling a better understanding of its suitability for particular use cases. By analyzing the transistor’s behavior in different application areas, we gain insight into its strengths, limitations, and strategies to overcome any performance challenges. This categorization approach can aid in the development of specialized and efficient PFETs tailored to meet the specific needs of different application fields, which is particularly useful in a general review of paper-based electronics.

### 3.1. PFETs for Sensing Applications

**Figure 10:**
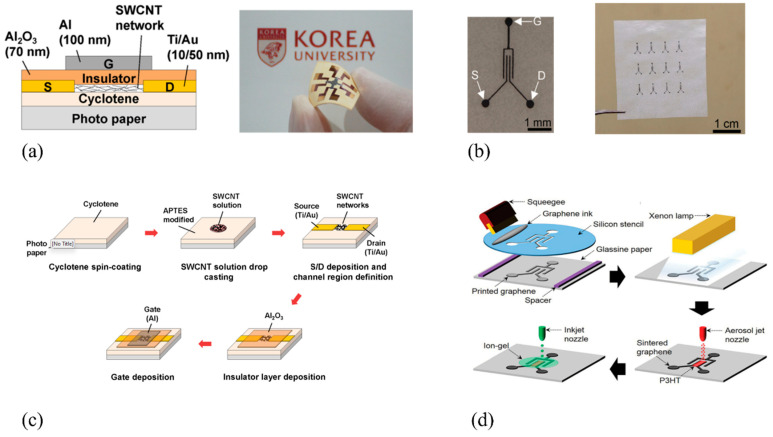
Schematic diagram, photograph, and fabrication procedure of (**a**,**c**) SWCNT TFT with Ti/Au contacts on paper substrate (reproduced with permission from [182], copyright 2015, AIP Publishing); (**b**,**d**) OTFT with graphene contacts on a glassine paper substrate (reproduced with permission from [183], copyright 2015, Wiley).

Paper-based FETs offer the advantage of being environmentally safe, disposable, and biocompatible, making them ideal for flexible electronics in wearable sensors, biomedical devices, smart cards, and other emerging applications. Various research groups have developed multipurpose PFETs using innovative techniques, leading to a significantly improved performance, which may potentially replace conventional FETs in a broad range of applications, while other works have focused on developing PFETs specialized for specific sensing applications. Figure 10 depicts the schematics of different TFTs with different fabrication strategies to highlight possibilities in device microfabrication and the compatibility of paper substrate with a few fabrication techniques.

Chuan Qian et al. [181] presented a novel method for developing flexible organic PFETs using cellulose paper as the substrate and the corresponding transistors demonstrated decent performance characteristics. The innovative aspect of this fabrication method is the reusability of the ion-gel dielectric, which can be peeled off and applied to new samples without a significant degradation in performance. The devices exhibit high mechanical flexibility and a good switching performance, positioning them as candidates for wearables and other disposable electronic applications. In another work [182], researchers made the initial effort to produce thin-film transistors (TFTs) using single-walled carbon nanotubes (SWCNTs) on commercially available photo paper. The excellent performance of these devices indicates their potential as a viable option for smart packaging and various sensing applications (Figure 10a–c). Another group [183] has presented a foldable organic TFT using untreated paper as a substrate. The device was created using various printing techniques, and a side-gate architecture was employed. The graphene electrodes used in the device demonstrated outstanding mechanical properties, while the ion-gel gate dielectric was highly deformable, allowing the device to endure bending. These characteristics, along with the devices’ low cost, render them ideal for use in foldable electronics, which play a critical role in wearable sensing applications (Figure 10b–d).

**Figure 11:**
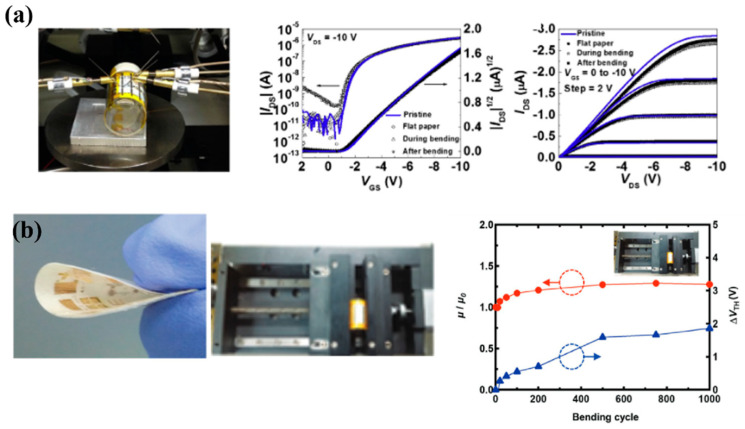
(**a**) TIPS-pentacene/PTAA OFET (reproduced with permission from [180], copyright 2017, Elsevier); (**b**) DNTT OFET under bending conditions (reproduced with permission from [189], copyright 2019, Wiley). The graphs beside each device indicate its mechanical stability under bending, showing minimal change in electrical performance.

Device and substrate flexibility is an important parameter because it allows applications in non-planar surfaces and more challenging installation sites that traditional rigid substrates. Paper offers good flexibility, and various researchers have conducted related measurements of the impact of mechanical deformation on device performance. Figure 11 depicts the bending properties of different PFETs and the graphs that indicate their mechanical stability. Shuanghe Jiang et al. [184] reported the development of an innovative type of low-voltage thin-film transistor (TFT) device using indium zinc oxide (IZO) and beeswax as the gate dielectric. The use of beeswax as a gate dielectric enabled the device to exhibit a high specific capacitance due to the electric double layer effect, while maintaining performance characteristics comparable to those of other paper-based TFTs. Furthermore, the non-toxic organic nature of the gate dielectric makes this device an ideal candidate for use in human-body-related sensors. In [180], the researchers presented their findings on an organic top-gate PFET that demonstrated improved mechanical flexibility and performance. They achieved this by using a CYTOP/Al_2_O_3_/nanolaminate gate dielectric layer on a paper substrate that was coated with a solution-processed polyvinyl alcohol/PVP buffer layer to fabricate TIPS-pentacene/PTAA-based top-gate OFETs (Figure 11a). The resulting OFETs exhibited high mobility and stability even after undergoing numerous bending cycles and prolonged operation. The reason for incorporating mechanical stability in the study was to evaluate the potential of the OFETs for wearable and flexible electronic applications where the devices must be capable of withstanding bending and other deformations without sacrificing their functionality or performance. Another group of researchers [175] have developed a new technique for producing polycrystalline silicon transistors on a cellulose paper substrate using a solution-based approach. The method involves depositing poly-Si material on the paper substrate and implementation of lithography for patterning. The resulting transistors exhibited a good electrical performance, comparable to those produced using traditional deposition methods. Additionally, the group implemented a blanket oxide etching technique to improve the performance of n-type TFTs, surpassing organic devices and competing IGZO TFTs. The study highlights the potential of this approach as a promising alternative to expensive and hard-to-find materials such as ln, especially in p-type devices. Taking advantage of their stable performance and their environmentally conscious design, these devices offer a scalable path toward applications such as smart packaging, biodegradable health monitoring systems, flexible displays, and other sensor nodes. Huixuan Liu et al. [185] have demonstrated the feasibility of flexible transistors made of indium oxide (In_2_O_3_) nanowires on a paper substrate. The nanowires were grown using the vapor–liquid–solid growth mechanism and used to fabricate paper-based nanowire transistors. These transistors were gated by a microporous SiO_2_-based electrolyte film, a technique [186] that has been previously used on other substrates such as glass [187]. The resulting devices showed an excellent electrical performance with high mobility values and low operating voltages, making nanowires a promising candidate for low-cost, high-performance, battery-powered PFETs for sensing applications. In a related work [188], an attempt was made to develop PFETs using graphene and MoS_2_ on glossy paper substrates. The RF graphene-based FETs exhibited high-frequency performance, with an intrinsic cut-off frequency of 25 GHz, making it a suitable candidate for disposable wireless sensing systems and other IoT applications. The MoS_2_ FETs showed a comparable DC and RF performance, with a high carrier mobility and I_ON_/I_OFF_ ratio, as well as a power gain of 7.2 GHz. Both devices were tested for their mechanical flexibility and demonstrated a stable electrical performance after undergoing thousands of bending cycles in a reasonable bending range. These findings highlight the potential of using paper substrates for the development of flexible and high-frequency, high-mobility monolayer FETs for various applications. In a recent research article [189], a new method for creating highly stable OFETs on paper was proposed. The technique involves a simple and universal surface planarization method, inspired by nanoimprint lithography technology, which allows the deposition of organic semiconductor layers with high uniformity and smoothness on a paper substrate. Through the fabrication of OFETs on paper, the researchers demonstrated the effectiveness of their approach by achieving high performance and improved stability over time, comparable to similar devices on silicon substrates. The stability of these OFETs was also confirmed through experiments testing their operational and mechanical durability, including excessive gate bias stress, and bending tests (Figure 11b).

**Figure 12:**
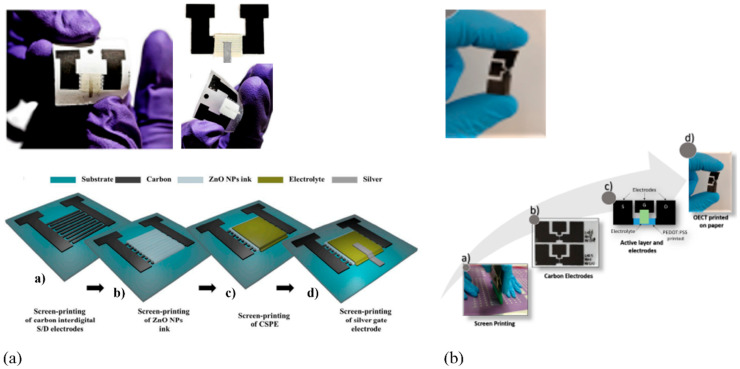
(**a**) Schematic representation of the fabrication steps and photograph of a fully printed ZnO EGT (reproduced with permission from [190], copyright 2019, MDPI); (**b**) inkjet-printed PEDOT/PSS-based OECT (reproduced with permission from [191], copyright 2021, IOP Publishing).

In their study, José Tiago Carvalho et al. [190] introduced an innovative method to produce transistors that are both low-cost and environmentally friendly, while also offering flexibility, using paper as a substrate. The authors utilized a specialized printing process to deposit a layer of carbon, followed by layers of zinc oxide, electrolyte, and silver onto the paper. These layers functioned cooperatively to generate n-type behavior, resulting in fully functional transistor devices that demonstrated satisfactory performance and stability under standard operating conditions. This approach enables the integration of inorganic semiconductors with printable electrolytes, tuned for roll-to-roll manufacturing, to fabricate flexible, paper-based transistors suited for applications such as biosensors, smart packaging, and disposable electronics. In another research article [191], the development of fully printed electrochemical transistors is described using PEDOT/PSS and inkjet technology on three different paper substrates. The study examines how the electrical properties of the resulting transistors are affected by three different paper surface types characterized by varying degrees of roughness. The findings reveal that the surface roughness of the paper substrate has a significant impact on the transistor’s electrical characteristics, such as its mobility and transconductance. The devices demonstrated impressive performance characteristics, including high I_ON_/I_OFF_ ratios, high transconductance, and low hysteresis, indicating their suitability for a wide range of sensing applications. Figure 12 displays photographs of two fully printed devices, accompanied by schematic diagrams outlining their step-by-step fabrication processes. These examples demonstrate how key printing techniques—specifically inkjet and screen printing—enable the fabrication of flexible, PFETs with reliable performance, well-suited for portable sensing and low-power electronic applications.

#### 3.1.1. PFETs as Biosensors

**Figure 13:**
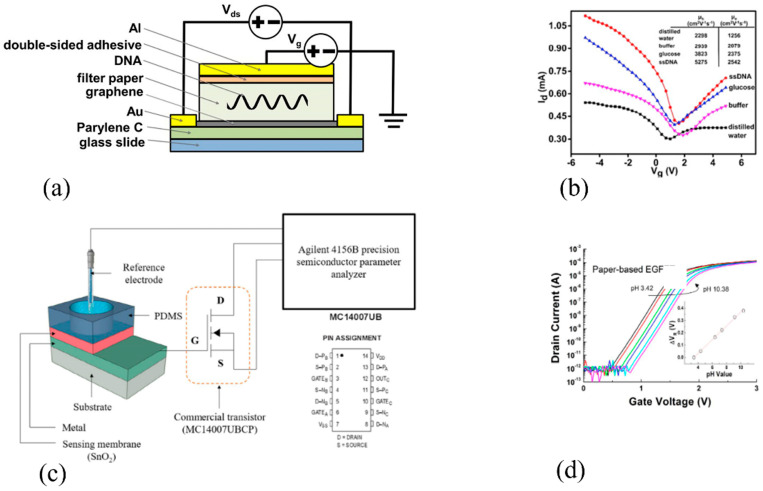
Schematic representation and characteristic curves of (**a**,**b**) paper-based GFET (Glucose and ssDNA sensor) (reproduced with permission from [192], copyright 2016, Elsevier); (**c**,**d**) paper-based EGFET device (pH sensor) (reproduced with permission from [170], copyright 2018, Elsevier).

PFETs have been the subject of several studies exploring their potential use as biosensors. A key advantage of PFETs is that their sensing layer can be engineered to detect specific biological molecules or target analytes with high sensitivity and selectivity. This sensing layer can be composed of a range of materials, including enzymes, antibodies, or other bioreceptors, which can interact with the target analyte and produce a measurable signal. The paper-based graphene-based field-effect transistor [GFET] developed by Aldrine Abenoja Cagang et al. [192] represents a simple and cost-effective approach to biosensing. The use of paper as a dielectric material enables the device to be easily and inexpensively fabricated using commonly available materials. The addition of specific molecules such as ssDNA or glucose to the two-dimensional paper network (2DPN) configuration increases the capacitance of the device, resulting in a measurable change in the current flowing through the device that is correlated with the concentration of the analyte. The results of this study provide valuable insights into the performance of GFET devices using paper-based substrates and offer a promising approach to the development of low-cost and portable biosensing devices. In another work [170], a paper-based extended-gate ion-sensitive field-effect transistor (EGFET) device is presented for pH-sensing applications, which offers a cost-effective solution due to its simple fabrication process. The device’s sensing properties are compared with those of EGFETs fabricated on silicon, glass, and PI substrates. The results showed that the paper EGFET exhibits a similarly high sensitivity to the other substrates, making it a promising option for practical sensing applications. The schematic diagram of two distinct PFET devices, as well as their characteristic curves, are illustrated in Figure 13.

#### 3.1.2. PFETS as Physical Sensors

**Figure 14:**
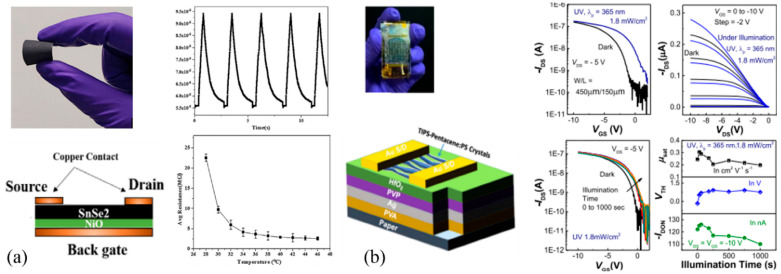
Digital image of fabricated devices, schematic representation, and characteristic curves of (**a**) paper-based MISFET (temperature sensor and photo switch) (reproduced with permission from [193], copyright 2020, Elsevier); (**b**) paper-based solution-processed OFET device (phototransistor) (reproduced with permission from [192], copyright 2016, Elsevier).

PFETs have emerged as a highly promising component for physical sensor applications, offering significant potential in diverse domains. These transistors exhibit the ability to provide a wide range of physical sensors, including chemical sensing, gas sensing, strain sensing, tactile sensing, temperature sensing, and optical sensing. The incorporation of tailored sensing materials or functionalization techniques empowers PFETs to selectively detect specific target analytes or respond to physical stimuli, enabling their deployment in several sectors such as environmental monitoring, healthcare diagnostics, and food quality control. Ongoing research in this field aims to further enhance their performance, sensitivity, and integration with advanced signal processing techniques, setting the stage for innovative applications in the field of physical sensing. Figure 14 illustrates the images, schematic diagrams, and characteristic curves of two PFET devices employed as versatile temperature sensors, photo switches, and phototransistors.

Sushmitha Veeralingam et al. [193] introduced a novel MISFET paper-based device that combined SnSe_2_ nanoflakes and a NiO gate insulator on paper. The back-end configuration, along with the incorporation of SnSe_2_ as the sensing layer, allowed the device to function as a photo switch. By adjusting the gate voltage, the device could be switched between different states based on the intensity of incident light. Furthermore, the device demonstrated excellent temperature-sensing capabilities, as the electrical conductivity of the SnSe_2_ nanoflakes was highly sensitive to temperature changes. As a result, the PFET device could serve as a multifunctional sensor with promising applications in light sensing and temperature monitoring. Figure 14a depicts the device and the characteristic curves of this work. It can be observed that simple material stacks on paper can produce devices with reliable performance and repeatability. In another study [194], a solution-processed organic field-effect transistor (OFET) implemented on paper substrates is introduced. This device exhibits remarkable performance and stability. In particular, the device demonstrates an excellent shelf life, resilience to humidity, and exceptional thermal properties, further enhancing its suitability for practical use. Additionally, this study explores the integration of novel paper-based phototransistors using the developed OFET platform. The phototransistors exhibit the ability to detect and respond to varying levels of incident light, making them highly valuable in light-sensing applications (Figure 14b).

**Figure 15:**
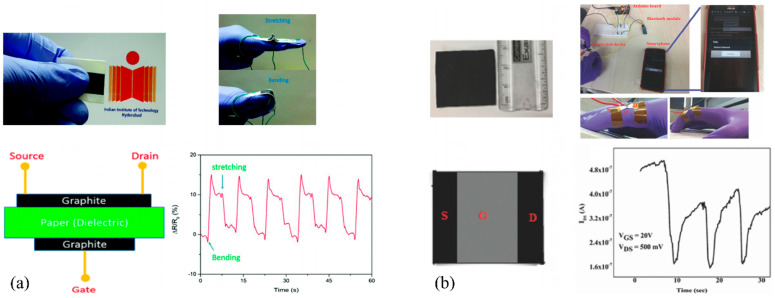
Demonstration of two different methodologies for integrating PFETs into devices designed for human motion detection: (**a**) carbon-based paper PFETs (reproduced with permission from [173], copyright 2016, Royal Society of Chemistry); (**b**) solution-processed graphene–MoS_2_ (Gr/MoS_2_) PFETs (reproduced with permission from [172], copyright 2018, Wiley).

Due to their remarkable mechanical stability and impressive resistance to bending, several PFET devices have garnered interest for potential applications in stress–strain sensing. Srinivasulu Kanaparthi et al. [173] presented a novel carbon-based PFET utilizing filter cellulose paper as both substrate and dielectric material. The fabrication process employed entirely sustainable techniques and materials, including the use of pencil graphite. Through experimental testing, the devices demonstrated remarkable sensitivity and stability as sensors for human motion detection. Another article [172] discusses the development of a wireless and smart system for human motion monitoring using Gr/MoS_2_ transistors fabricated on paper substrates through a solution-based process. The incorporation of graphene as a semiconductor played a dual role by increasing the mobility of charge carriers and forming a potential barrier with MoS_2_. This unique combination resulted in enhanced sensitivity towards small strains. To highlight the capabilities of the sensor, it was integrated onto the human hand, allowing wireless monitoring of hand movements. The collected data were transmitted wirelessly and monitored using a smartphone, providing a convenient and portable solution for tracking human motion. Another study [195] aimed at developing low-voltage transistors using paper substrates, and ion gel and cellulose fiber composites were employed as key materials. By utilizing the benefits of additive manufacturing techniques for semiconductors and conductors, the authors were able to construct flexible transistors on paper substrates that exhibited impressive performance while being easy to fabricate. These transistors have the potential to be used for various sensing readout applications, as exemplified by the tactile sensing surface presented in the study. Figure 15 presents two distinct approaches for integrating PFETs into stress–strain-sensing devices. A comprehensive list of various PFETs is presented in Table 4, where the key factors such as materials used and performance metrics are listed.

### 3.2. PFETs—Analog-Digital Circuits/Memories

Paper-based integrated circuits (ICs) have attracted considerable scientific attention due to their eco-friendly characteristics and remarkable progress in recent studies [8,10,196]. ICs utilize transistors, resistors, capacitors, and interconnecting metal traces as fundamental components to construct logic gates, inverters, memories, flip-flops, multiplexers, and other essential elements for digital circuits. Researchers have made significant efforts to develop paper electronic circuits, employing PFETs as the fundamental building block.

**Figure 16:**
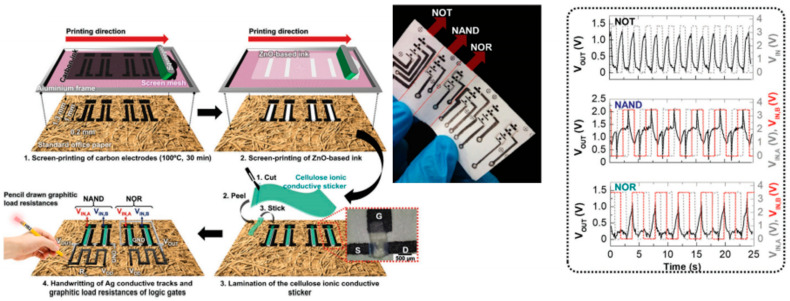
Screen-printed ZnO-based electrolyte-gated transistors (EGTs). The illustrations highlight the step-by-step printing process including gate, source, drain, and semiconductor deposition. Real-time electrical measurements demonstrate the functional performance of NOT, NAND, and NOR logic gates (reproduced with permission from [47], copyright 2021, Wiley).

Demonstrating basic logic gates (e.g., AND, OR, NAND, NOR) validate that paper-based transistors can be reliably interconnected to execute digital logic operations. It also confirms the capability of the devices for multi-transistor integration, the next step toward the development of scalable logic circuits. Once logic gate functionality is established, more complex digital architectures such as counters, encoders, multiplexers, and memory elements could be designed. In 2017, Kalyan Yoti Mitra and colleagues [197] introduced an innovative method for fabricating thin-film transistor (TFT) arrays on paper substrates using a completely inkjet-printed process. This work marked the first comprehensive publication detailing the fabrication of fully inkjet-printed organic transistors on paper. The technique involved depositing conductive, semiconductive, and dielectric materials as inks onto specially coated paper, resulting in TFTs that exhibited promising electrical characteristics, such as charge carrier mobility and on/off ratios indicative of an effective switching performance. The entire fabrication process utilized commercially available substrates and inks with predefined properties, ensuring the method’s reliability and scalability. Another study [198] explores the development of functional logic gates for digital circuit applications using inkjet-printed single-walled carbon nanotube (SWCNT) field-effect transistors (FETs). The devices were fabricated on a photo paper substrate, demonstrating the feasibility of disposable, low-cost electronic circuits. The research focuses on optimizing SWCNT-based PMOS transistors to enhance circuit performance. Future advancements in CNT doping techniques and ink formulation could further improve device efficiency, making them viable for commercial applications. Cunha et al. [47] have developed screen-printed ZnO electrolyte-gated transistors (EGTs) on standard office paper, successfully integrating NOT, NOR, and NAND logic gates using handwritten conductive paths with graphitic load resistances. This study introduces a water-based, screen-printable ZnO nanoparticle ink, enabling low-temperature fabrication without the need for sintering, ensuring compatibility with standard paper substrates. The transistors utilize a cellulose-based ionic conductive sticker as a gate material, enabling sub-2.5 V operation with good electrical performance even under bending conditions. This makes them highly suitable as fundamental building blocks for the development of more complex, flexible electronic circuits. Figure 16 highlights the integration of the paper-based transistors with functional logic gates.

**Figure 17:**
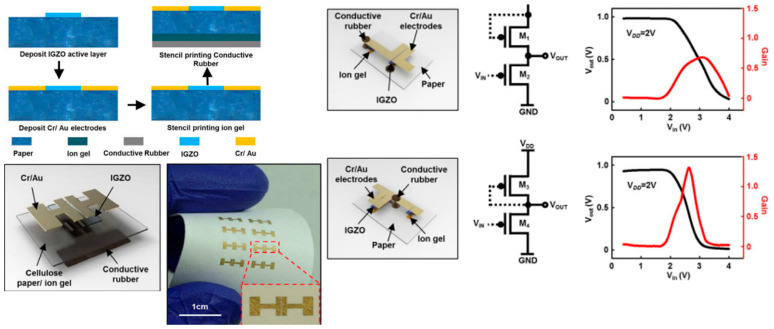
Fabrication process, optical images, schematic structures, and voltage transfer characteristics of inverter circuits based on paper-based printed IGZO TFTs (reproduced with permission from [195], copyright 2020, Springer Nature).

The inverter (NOT gate) is the simplest and most fundamental building block in digital electronics. An inverter circuit produces an output that is the logical opposite of its input. Its primary function is to reverse the input signal. It enables the evaluation of voltage transfer characteristics (VTC), providing key insights into gain, noise margins, and switching thresholds. Also, implementing inverters using unipolar or complementary transistors helps to assess the device symmetry, drive strength, and load matching. Since paper electronics target low-power and low-voltage applications, inverters help to evaluate whether the transistors can switch cleanly at minimal voltages, ensuring energy efficiency. Wang et al. [199] have developed a novel method for producing flexible, low-voltage TFTs by incorporating a graphene oxide-enhanced poly(vinyl alcohol) (PVA) film as the gate dielectric. This innovative approach enables the TFTs to operate efficiently at low gate voltages while exhibiting exceptional stability during bending, due to the reinforcing properties of graphene oxide. Additionally, the researchers explored the development of a resistor-loaded inverter and assessed its dynamic response. The findings demonstrated that the inverter’s effective switching between high and low states in reaction to the input signal, highlighting its potential for digital circuits and flexible electronics applications. Another study [195] introduces a paper-based TFT in which the paper functions not only as a substrate but also as an active dielectric component. By infusing cellulose fibers with ion-gel composites, the device achieves low-voltage operation (~1.8 V), effectively overcoming the challenge of high operational voltage in conventional paper transistors. The transistor was integrated into digital logic circuits, including two types of inverters and active matrix multiplexing arrays, both demonstrating efficient voltage transfer. Additionally, the ion-gel/cellulose dielectric transistor array successfully functioned as a multiplexing readout circuit, displaying its potential for several electronics applications. The physical sensing capabilities of the device have been previously discussed in the earlier section. Raghuwanshi et al. [200] introduced a novel approach to organic field-effect transistors (OFETs) on paper, utilizing a hybrid bilayer dielectric of HfO_2_ and PVA and a TIPS-pentacene/PS blend as the semiconductor material. This design enables low-voltage operation, enhancing device performance and stability. Additionally, the study successfully demonstrated a resistive-load inverter with variable resistance, showcasing its potential for printed logic circuits. Figure 17 highlights the implementation of functional inverters utilizing the unique properties of PFETs.

**Figure 18:**
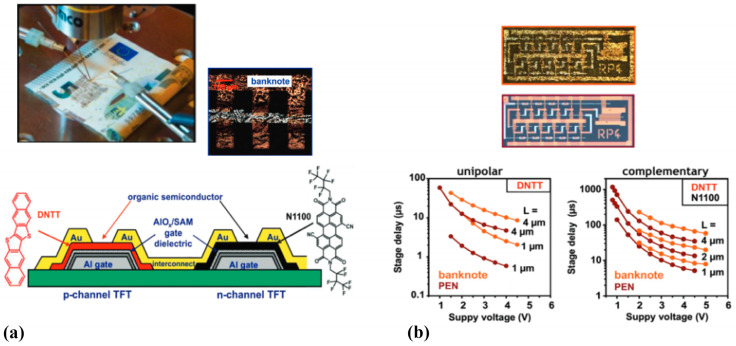
(**a**) Photograph of a banknote with integrated organic TFTs and circuits alongside schematic cross-section of the TFT structure; (**b**) photographs of unipolar ring oscillators and signal delay per stage in 11-stage unipolar and complementary ring oscillator circuits (reproduced with permission from [48], copyright 2019, Wiley).

A ring oscillator is a circuit made up of an odd number of inverters (NOT gates) connected in a loop, where the output continuously oscillates between high and low voltage levels, representing logical true and false. The output of the final inverter is fed back into the first, creating a self-sustained oscillation. Ring oscillators are valuable tools for converting static transistor behavior into time-domain signals, allowing precise measurement of signal delay, switching speed, and frequency response. Their successful fabrication demonstrates that transistors can operate reliably within integrated circuits, beyond isolated, static performance tests. Pettersson et al. [201] developed eco-friendly, low-voltage ion-modulated transistors (IMTs) on paper substrates by combining a biodegradable polymer–semiconductor blend with deep eutectic ionic liquids as the electrolyte. This design enhanced device performance, particularly in switching behavior, and enabled the realization of functional printed logic circuits. Among the circuits’ fabricated ring oscillators, NOR gates, and SR latches, the ring oscillators were especially significant, highlighting a dynamic performance with a frequency of ~1 Hz and a stage delay of ~100 ms. Their successful operation demonstrated that IMTs can support both static logic functions and dynamic, time-dependent signal processing. Another study [48] displays the development and analysis of low-voltage, high-frequency organic transistors and both unipolar and complementary ring oscillators, innovatively fabricated on banknote surfaces. It provides a detailed comparison between TFTs created on banknotes and those on traditional substrates like glass and PEN, emphasizing the impact of substrate surface roughness on the transistors’ electrical performance. The research highlights the ability of these paper-based organic integrated circuits to operate at supply voltages below 3 V and achieve frequencies in the range of several hundred kilohertz. Importantly, the use of complementary ring oscillators, which include both p-channel and n-channel transistors, is underscored for their role in minimizing power consumption and enhancing noise margins. Although the study observed that smoother substrates like glass and PEN led to reduced signal delays, the banknote-based ring oscillators still demonstrated lower supply voltage requirements and smaller signal delays compared to prior research, signifying advancements in the field of paper-based electronic devices. Figure 18 depicts integrated organic TFTs used in unipolar and complementary ring oscillator circuits.

**Figure 19:**
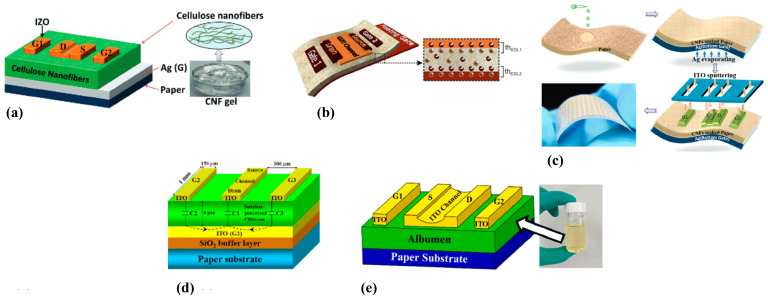
(**a**) Three-dimensional schematic of an in-plane double-gate EDLT using a CNF film as the gate dielectric (reproduced with permission from [202], copyright 2017, Wiley); (**b**) PDG/BFG-FET with floating back gate based on IZO conductive film (reproduced with permission from [203], copyright 2018, Elsevier); (**c**) ITO-based TFT with CNFs-processed paper gate dielectric (reproduced with permission from [204], copyright 2019, Wiley); (**d**) junctionless dual-gate paper TFT (DGTFT) (reproduced with permission from [206], copyright 2019, American Chemical Society); (**e**) dual-gate planar laterally coupled TFT using egg albumen-based dielectric (reproduced with permission from [207], copyright 2021, Elsevier).

A notable trend in recent studies is the adoption of multi-gate architectures, including dual-gate and in-plane gate configurations, which enhance device functionality by offering improved electrostatic control, tunable threshold voltages, and greater logic programmability. The paper by Feng Shao et al. [202] presents an innovative approach to fabricating multi-gate oxide-based electric-double-layer transistors (EDLTs) on paper substrates using cellulose nanofibers (CNFs) as the gate dielectric. This approach highlighted the superior electrical performance because of EDL coupling, notably achieving universal NAND logic using a single transistor with a resistor. These devices represent an important advance in the field of paper-based digital circuits, especially in applications where high-speed operation is secondary. Another group of researchers [203] have developed a planar double-gate indium gallium zinc oxide (IGZO) FET featuring a back floating gate electrode (PDG/BFG-FET), utilizing paper for both substrate and dielectric functions. This configuration allows the device to execute multiple logic operations (including NOT, OR, AND, NOR, NAND gates) through the simple modulation of the amplitude and frequency of the input gate signals. The device operates with low power consumption while demonstrating a notable on/off ratio and mobility. Another approach [204] presents novel low-voltage flexible TFTs fabricated on cellulose nanofiber (CNF)-soaked paper, which functions simultaneously as the substrate and gate dielectric. These transistors employ indium tin oxide (ITO) in a homojunction configuration for both the channel and electrodes and feature dual in-plane gates that enable the implementation of advanced logic functions such as inverters and NAND gates. Another indium tin oxide (ITO)-TFT device featuring two lateral in-plane gates is presented by Nie et al. [205], utilizing an ionic liquid/chitosan-coated paper, where the paper simultaneously functions as both the substrate and gate dielectric. The incorporation of an ionic liquid/chitosan composite provides a high electric double-layer (EDL) capacitance, enabling low-voltage operation. To demonstrate circuit functionality, both a NAND gate and an inverter were successfully implemented. Another study [206] introduces a junctionless dual-gate thin-film transistor (DGTFT) fabricated entirely on paper substrates, also using chitosan-based solution-processed dielectrics and indium tin oxide (ITO) for both the channel and the electrodes. The device utilizes dual in-plane gate electrodes, allowing for threshold voltage modulation and the implementation of an AND logic function using a single transistor, significantly simplifying circuit design. Finally, Guo et al. [207] demonstrated the use of untreated egg white, dried at room temperature to preserve its ionic conductivity, as a solid electrolyte dielectric, combined with indium tin oxide (ITO) as the semiconductor to fabricate in-plane thin-film transistors (TFTs). These devices were successfully applied to implement AND and NOT logic gates using resistor-loaded inverters, exhibiting stable electrical performance and strong noise immunity. Figure 19 presents schematic illustrations of various transistors featuring dual-gate and in-plane gate configurations, highlighting the structural differences and gating strategies.

Memories in integrated circuits (ICs) come in several types, including those based on capacitors, resistors, or transistors. These memory transistors, often referred to as memory cells, serve as the fundamental building blocks of memory circuits and are indispensable components within ICs. Memory devices can be categorized as either nonvolatile or volatile. Nonvolatile memory retains stored information even without power, whereas volatile memory loses its data when power is turned off. Noteworthy progress has been made in the development of flexible transistors, particularly for their potential use as memory devices [208,209,210]. In the last decade, several PFETs have been successfully demonstrated as memory components, offering the prospect of eco-friendly and sustainable memory solutions.

**Figure 20:**
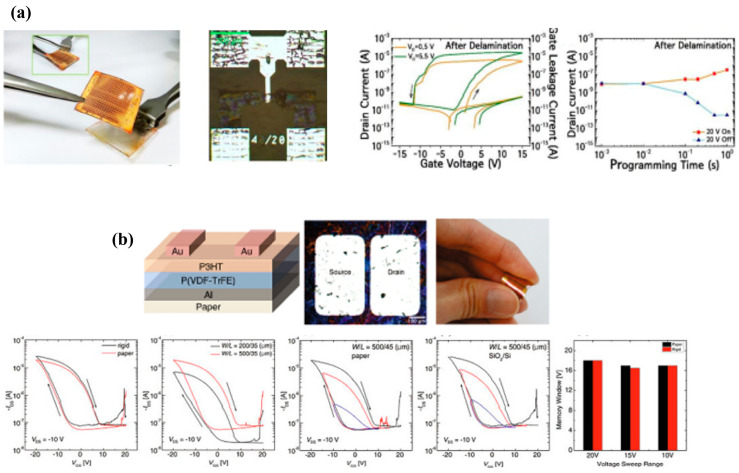
(**a**) Photographs, schematic diagrams, and electrical performance of a chicken albumen gate insulator TFT (reproduced with permission from [49], copyright 2016, Royal Society of Chemistry). (**b**) Ferroelectric OFET memory on paper substrates (reproduced with permission from [212], copyright 2020, Korean Physical Society).

In a study by So-Jung Kim et al. [49], a novel approach to eco-friendly TFTs was introduced, employing egg albumen as the dielectric material. These TFTs demonstrated promising potential for use as memory transistors with programming capabilities. The devices exhibited reliable transistor characteristics, including high mobility and a high on/off ratio. Additionally, the researchers exploited the switching phenomenon of residual polarization in the albumen, enabling the TFTs to function as nonvolatile memory devices. Xu et al. [211] presented a multi-bit organic field-effect transistor (OFET) memory on a commercial paper substrate. The device leverages a tri-layer ferroelectric gate dielectric architecture to enable low-voltage, stable, and programmable multi-level memory within a single transistor. This structure allows precise control over remnant polarization and charge trapping, facilitating a 2-bit (four-level) nonvolatile memory operation. Another group [212] developed a ferroelectric field-effect transistor (FeFET) using a solution-based process, without the need for protective layers or lithography. The device exhibited clear nonvolatile memory behavior, with a ~16 V memory window. Compared to transistors on conventional SiO_2_/Si substrates, the memory performance on paper was comparable, confirming its suitability for flexible, low-cost memory applications. Figure 20 illustrates two distinct approaches for fabricating memory transistor devices on paper substrates, highlighting differences in device architecture, materials, and performance characteristics. Table 5 includes a comprehensive overview of PFETs used as memory and logic devices, with a detailed presentation of each critical parameter, alongside with materials and fabrication techniques.

## 4. Detection Techniques in Paper-Based Sensors

Paper-based sensors utilize a wide range of detection principles depending on the nature of the analyte and the intended application. These techniques fall broadly into electrical, optical, and electrochemical modalities, with recent developments enabling hybrid and smartphone-assisted readouts. This section compiles the key detection strategies employed in paper-based sensors, emphasizing their operating principles, outputs, and use cases. Table 6 presents an overview of each detection mechanism, alongside with recent examples taken from corresponding publications.

### 4.1. Electrical Detection

Electrical detection techniques involve the measurement of changes in resistance, capacitance, or current in response to a physical or chemical stimulus. Common examples include resistive humidity sensors and capacitive strain sensors. Field-effect transistor (FET)-based paper sensors also fall within this category, where the interaction between the target analyte and the semiconducting channel leads to modulation of the source-drain current, providing high sensitivity and rapid response.

### 4.2. Optical Detection

Optical detection mechanisms include colorimetric and fluorescence-based sensing. These methods provide a visual output, often visible to the naked eye or via smartphone imaging systems. Colorimetric paper sensors are widely used for detecting heavy metals, pesticides, and pathogens through observable color changes triggered by chemical reactions. Fluorescence-based sensors typically involve the use of nanomaterials or labeled bioreceptors that emit light upon excitation, allowing for sensitive detection of trace analytes such as cancer biomarkers or environmental toxins.

### 4.3. Electrochemical Detection

Electrochemical sensors rely on redox reactions occurring at printed electrodes on the paper substrate. These devices commonly use screen-printed carbon, silver, or gold electrodes and are integrated with functional materials such as enzymes, nanoparticles, or conductive polymers. Techniques such as cyclic voltammetry, differential pulse voltammetry, and amperometry are employed to quantify analytes based on the electrical signals generated. Electrochemical paper-based sensors are extensively used in glucose monitoring, VOC detection, and heavy metal sensing.

### 4.4. Hybrid and Integrated Approaches

Recent advancements have combined optical and electrochemical mechanisms in dual-mode sensors, improving accuracy and reliability. Furthermore, energy-autonomous platforms, such as those incorporating triboelectric nanogenerators, are being explored to eliminate the need for external power. Integration with wireless modules and mobile applications is also expanding the scope of paper-based sensors for decentralized and wearable diagnostics.

## 5. Conclusions—Future Outlook

This review paper focused on gathering, analyzing, and presenting information on sensors and PFETs fabricated onto paper substrate. We divided the manuscript into two main categories, namely sensors and PFETs. For each category, we have a dedicated analysis of the most important recent works, comprising figures, materials used, performance metrics, and fabrication techniques. This application-wise categorization was chosen for this article so it can act as a guide for future paper-based-device development.

Sensors include physical sensors (Table 1) such as UV, temperature, relative humidity, strain, and pressure. These sensors on paper fabrication strategies expand from printing [66,69,71,73,83], to LIG [65] and self-assembly of the materials [60,67,68], to spray-coating [70,78], and even handwriting of the pattern [63,79]. The materials that have been used are diverse; carbon [58,59,62,67,69,73,77,79,81,83] and graphene-based materials [45,56,58,60,61,64,65] have the most frequent references, mainly because they are suitable for facile electrode development.

Paper-based electrochemical sensors (Table 2) for detecting volatile organic compounds and heavy metals have been presented; these sensors work with either electrical [88,90,95,96,97] or optical [98,100,104] output and can detect a wide range of harmful substances. Given the high specificity of the target analytes, this category of sensors is indicative of the capabilities of paper to act as a substrate for an exceptionally large application field, where the deposition of active materials in the same substrate can create multi-response portable tests [96,97,102].

Biocompatibility is a major advantage of paper-based sensors. Electronics developed on cellulose-based substrates can inherit this property, which is a key reason for their widespread use in the development of biodevices. In this review, we have included two dedicated sectors for biodevices; in the first part we discuss biosensors, where mostly passive sensors are used as point-of-case systems. More specifically, we analyzed recent paper-based glucose sensors (Table 3) and concluded that simple processes such as drop casting [121,124,138] coupled with a printing process are adequate to create cost-effective glucose-sensing solutions. More complex processes such as CVD [115], electrodeposition [116,118,123], sputtering [115] and more, highlight paper’s compatibility with such technologies, so if needed, more accurate devices can be produced. The second category of biodevices includes active devices such as bio-FETs, where a physical bio-signal can directly modulate a FET electrical parameter [192]; therefore, various quantities can be measured, such as pH [170], glucose, and ssDNA [192].

The development of PFETs using innovative techniques, leading to significantly improved performance, may potentially replace conventional FETs in a broad range of applications such as sensors. Table 4 highlights the fact that these devices could sense a wide range of signals by selecting suitable semiconductors, electrolytes, or functional coatings. A wide variety of semiconductors, e.g., organic molecules such as P3HT [181,183], carbon nanomaterials such as SWCNTs [182] or graphene [192], metal oxides such as IZO and ZnO [184,190,195], and even 2D materials such as MoS_2_ [172,188] could be used. Dielectrics range from ion gels, polymer electrolytes, or other organic materials to inorganic oxides (Al_2_O_3_, SiO_2_) that can provide better stability but may require higher operation voltages. Reported carrier mobilities can vary from orders of magnitude (from ~10⁻^3^ cm^2^ V⁻^1^ s⁻^1^) in simpler designs to tens or even hundreds of cm^2^ V⁻^1^ s⁻^1^ for advanced devices. Threshold voltages similarly vary (from <1 V for ion-gel or electrolyte-gated designs to >30 V for more conventional oxide dielectrics). These FETs enable diverse sensing functionalities across multiple applications from physical sensors (strain gauges [172,173], temperature switches [193], optical/light sensors [194], and tactile sensors [195]) to biosensors (glucose, ssDNA [192], and pH [170]).

Unlike most sensing-oriented devices, Table 5 displays paper-based transistors used in higher-level circuit functions such as inverters, logic gates, oscillators, and even memories. Semiconductors span from organic (e.g., P3HT [212], DNTT [48], pentacene [211]) to inorganic oxides (e.g., IGZO [47,49,199,202,203], ITO [204,207]), and hybrid systems (blends or deep eutectic mixtures [192]). Dielectrics include classical oxides (Al_2_O_3_, HfO_2_, etc.), but also electrolyte-based or ionic gels (e.g., chicken albumen electrolytes, cellulose-based hydrogels). Carrier mobilities range from modest (~10⁻^2^–10⁻^1^ cm^2^V⁻^1^s⁻^1^) up to impressive values (>1 cm^2^V⁻^1^s⁻^1^) for optimized oxide or well-defined organic devices. Threshold voltages vary widely (from near 0 V to ~14 V) due to differences in the materials’ dielectric properties and device architectures. Some devices integrate dual-gate or multi-gate architectures to enhance their performance characteristics or enable memory functionality. Finally, multiple reports demonstrate more complex functional blocks that can be realized only using multi-transistor integration on paper, paving the way for fully integrated paper systems.

Paper-based printed electronics have emerged as a transformative approach in the development of sustainable, flexible, and cost-effective electronic devices and systems. Recent advances in functional materials, printing techniques, and device architectures have significantly expanded the capabilities of paper-based platforms, enabling applications in sensing, energy harvesting and storage, communication, and even integrated system-level functionalities. Despite remarkable progress, challenges remain in achieving long-term device stability, high-resolution printing, and scalable manufacturing. Future research will need to focus on several key directions. The development of novel, eco-friendly conductive and semiconducting inks with improved printability, durability, and compatibility with paper substrates is essential. Additionally, hybrid systems that combine paper electronics with complementary flexible or rigid platforms could bridge the gap between disposability and high performance. Integration with wireless communication modules and data processing units will be critical for enabling smart, connected systems in domains such as the Internet of Things (IoT), smart packaging, wearable healthcare, and environmental monitoring.

While paper-based devices are frequently praised for their sustainability and low-cost fabrication, a critical evaluation reveals several unresolved challenges that hinder their practical deployment. Despite their compatibility with additive manufacturing techniques and appeal in disposable applications, paper substrates inherently suffer from issues such as poor dimensional stability, moisture sensitivity, and surface irregularity. These factors significantly compromise the reproducibility and reliability of electronic performance. For instance, the integration of field-effect transistors (PFETs) on paper, while technologically impressive, remains academic; most reported devices exhibit limited carrier mobility, high variability, and poor long-term stability under ambient conditions. Moreover, claims of flexibility and biocompatibility are often offset by the need for extensive material modification, which in turn increases fabrication complexity and cost—undermining the original advantages of using paper. Compared to polymer or silicon platforms, paper-based devices currently fall short in achieving the consistent electrical characteristics required for logic applications or robust sensing. Without substantial advances in substrate engineering, encapsulation methods, and circuit-level integration, these devices are unlikely to move beyond laboratory-scale demonstrations. As such, while promising in concept, paper-based electronics remain an emergent technology in need of deeper standardization, rigorous benchmarking, and clearer pathways to real-world deployment. Table 7 provides a comparison of the key characteristics of paper-based devices.

As regulatory and sustainability considerations become increasingly important in electronics manufacturing, paper-based printed electronics are poised to play a vital role in the next generation of green technologies. Their evolution will depend on continued interdisciplinary collaboration across materials science, electronics, printing technology, and system integration. With these coordinated efforts, paper-based electronics can transition from laboratory-scale prototypes to practical, real-world solutions that align with both technological and environmental imperatives.

## Figures and Tables

**Table 1:** Paper-based physical sensors overview.

Type	Active Materials	Substrate	Range	Fabrication Method	Ref.
Relative humidity	Carbon black, exfoliated graphene	Filter paper	33–95%	Dip coating	[62]
	2B graphite, ethanol	White, non-glossy, hard cellulose	43–83%	Hand-drawn	[63]
	rGO/PANI	Polypropylene filter paper (0.22 μm)	0–98%	Filtering	[64]
	Graphene	Filter paper	10–98%	LIG	[65]
	Nanoporous SiO_2_–Al_2_O_3_	P:E smart paper, PEL P60	15–92%	Inkjet printing	[66]
	CNTs	(TEMPO)-oxidized cellulose fibers	11–95%	Self-assembly	[67]
	GO, Al	–	30–90%	Self-assembly, thermal evaporation	[68]
	Cellulose nanofiber/CNT	Custom PBHS with different weights	11–95%	Roll-to-roll	[69]
	Writing carbon ink	A4 paper (80 g m^−2^)	18–91%	Dipping, spraying	[70]
	EPTAC-cellulose and Ag electrodes		11–95%	Bath, stirring, and screen printing	[71]
	Ionic conductive WCN—TEMPO and Au		7–94%	Stirring, shadow mask	[72]
	MWCNTs–Ag	Porous paper (PPHS)	10–90%	Screen printing, gravure printing	[73]
	Al–PI–polyester conductive adhesive tape		41.1–91.5%	–	[74]
	Bacterial cellulose—CNTs	–	0–98%	Mayer rod	[75]
	Edible rice paper		0–100%	Drawing	[76]
Pressure	GO-cellulose composite paper		0–20 kPa	Thermal reduction	[56]
	Carbonized graphene-coated wastepaper aerogel		0.3–5 kPa	Stirring, molding, annealing	[58]
	rGO/PMMA porous structure/PI		0–2.5 MPa	Vacuum filtration	[59]
	Microporous, free-shaped reduced graphene oxide paper/PI		0–60 kPa	Evaporation-induced self-assembly	[60]
	Graphene/ZnO	Paper, cotton	0–100 kPa	Coating, in situ synthesis	[45]
	CNT	Printing paper	–	Elevated temperature pressing	[77]
	CNT/PI	Paper	8–140 kPa	Spray coating	[78]
	CNT/PDMS/PI	Tissue paper	0–42 kPa	Handwriting	[79]
	Chitosan/potato starch/PVA/FeCl_3_		0–250 kPa	Molding	[80]
Strain	Carbonized graphene-coated wastepaper aerogel		0–75%	Stirring, molding, annealing	[58]
	rGO/PMMA porous structure/PI		0–10%	Vacuum filtration	[59]
	Microporous, free-shaped reduced graphene oxide paper/PI		7.6–11%	Evaporation-induced self-assembly	[60]
	Graphene	Mulberry paper	0.28–0.58%	Meyer-rod coating	[61]
	MWCNT/PDMS	Photo paper	0–130^°^	Screen printing	[81]
	CNT/poly-m-phenylene isophthalamide (PMIA)		–	Hot-press	[82]
	MWCNT/PET	Paper	0–1.72%	Screen printing/lamination	[83]
	Graphene	Nanocellulose-based	0.55%	Scrape coating/deep coating	[84]
	CB/Graphene/SiO_2_	Sodium carboxymethyl	−1.0–1.0%	Dip coating	[85]
	Chitosan/Potato starch/PVA/FeCl_3_		200%	Molding	[80]

**Table 2:** Paper-based electrochemical sensors.

Active Materials	Principle	Target Analyte	LoD *	Ref.
Graphene-PEDOT/PSS, WS_2_	Electrical	Butanol	50 ppm	[88]
Gold, nanostructured latex-coated paper	Electrical, colorimetric	H_2_S	1.5 ppm	[89]
Carbon black	Electrical	Bisphenol A	0.03 μM	[90]
CdTe and ZnCdSe quantum dots—nanoporphyrins	Colorimetric	Organophosphorus (dimethoate)	1 μgL^−1^	[94]
Perovskite halide CH_3_NH_3_PbI_3_ (MAPI)	Electrical	NH_3_	<1 ppm	[95]
Graphene paper, Nafion, Bi nanoparticles	Electrical	Pb^2+^, Cd^2+^	0.1 ppb	[96]
GO, ZnO nanoparticles, EDTA	Electrical	Cd^2+^, Pb^2+^, Cu^2+^, Hg^2+^	1–6.8 μM	[97]
AgNPs	Colorimetric	Hg2+	10 µgL^−1^	[98]
Citrate capped Cu@Ag core–shell nanoparticles	Colorimetric	Phenthoate	50–200 μgL^−1^	[46]
γ-MnOOH nanowires	Colorimetric	Organophosphorus (AChE, omethoate)	0.1 mU mL^−1^, 10 ng mL^−1^	[100]
Quantum carbon dots, CdZnTe quantum dots	Fluorescence	Hg^2+^-Sulfide	0.002 and 1.488 μM	[101]
Belt-like ZnSe nanoframes	Colorimetric	Ag^+^, Cu2^+^, Hg^2+^	5 ppm, 1 ppm	[102]
Au@Ag NPs	Colorimetric	TNT	0.35 μg/mL	[103]
Colloidal gold labeled mice monoclonal antibody	Colorimetric	Toltrazuril	<2.60 μg/kg	[104]

* Limit of detection.

**Table 3:** Paper-based glucose sensors.

Active Materials	Range	LoD * (μΜ)	Output	Fabrication Method	Ref.
Graphene/Cu_2_O	0.5–5166 μM	0.21	Electrical	CVD-thermal decomposition	[115]
Graphene/PtCo alloy NPs	0.035–30 μM	5	Electrical	Electrochemical deposition	[116]
Graphene/CuO/Cu(OH)_2_	50 μM–10 mM	7	Electrical	Thermal-laser modification	[117]
rGO/Cu nanoflower	2 μM–13 mM	0.5	Electrical	Mold casting–electrochemical deposition	[118]
AuNPs/graphene paper	15 μM–8 mM	2.5	Electrical	Sputtering and thermal, laser dewetting	[119]
Carbon, cobalt phthalocyanine, graphene, ionic liquid	0.01–5.0 mM	0.67	Electrical	Wax printing, screen printing, drop casting	[120]
Aniline functionalized graphene quantum dots, PBA	0.05–20 mM	2.1	Optical	Inkjet, drop casting	[121]
Graphene nanosheets, carbon nanotubes, PtAu	0.1–11.6 mM	8.0	Electrical	Roll printing, electrodeposition	[123]
Activated carbon, Cu(II)	0.0004–7 mM	0.2	Electrical	Drop cast	[124]
Graphite powder, Cu(II)	0.00007–5 mM	0.05	Electrical	Drop cast	[124]
MWCNT-COOH, Cu(II)	0.00002–8 mM	0.02	Electrical	Drop cast	[124]
Cu(II)/MWCNT-COOH (1:5)	0.0003–9 mM	0.3	Electrical	Drop cast	[124]
rGO-TEPA/PB	0.1–25 mM	25	Electrical	Screen printing, photolithography	[126]
Prussian blue–graphene modified with GOx and chitosan	2–650 μM	–	Electrical	Wet spinning	[127]
Chitosan/GOx/horseradish peroxidase/TMB	0–250 μM	10	Optical	Wax soaking, drop cast	[136]
Fluorescent silicon nanodots/graphene	2.68–200 μM	–	Fluorescence	Laser engraving, LIG	[137]
Graphite/Ag/AgCl/graphene-COOH	5–500 pM	0.0015	Capacitive	Hand-drawing, drop cast	[138]
AuNP–AgNP	500–6.000 μM	340.0	Optical	Wax printing, drop cast	[135]
Ni–HHTP	–	1.30	Electrical	Screen printing, drop cast	[139]

* Limit of detection.

**Table 4:** Paper-based FETs for sensing applications.

I_on_/I_off_	Carier Mobility (cm^2^V^−1^S^−1^)	Threshold Voltage (V_TH_) (V)	Semiconductor	Dielectric	Type of Device	Applications	Ref.
~10^3^–10^4^	0.97	0.67	Poly(3-hexylthiophene) (P3HT)	Ion-gel dielectric	OFETs	Sensing applications	[181]
~10^6^	~3	-	Single-walled carbon nanotubes (SWCNTs)	Aluminum oxide (Al_2_O_3_)	TFTs	Sensing applications—smart packaging, attachable displays	[182]
2.8(±0.9) × 10^3^	0.14 ± 0.05	1.04 ± 0.15	Poly(3-hexylthiophene) (P3HT)	Ion-gel dielectric	OTFTs	Sensing applications—foldable electronics	[183]
7.6 × 10^6^	14.6	0.46	Indium zinc oxide (IZO)	Organic beeswax	TFTs	Sensing applications—portable electronics (Human body related sensors)	[184]
~10^5^	1.7 ± 1.1 10^−1^	1.4 ± 0.2	6,13-Bis(triisopropylsilylethynyl) pentacene (TIPS-pentacene) and Poly[bis(4- phenyl)(2,4,6-trimethylphenyl)amine] (PTAA) (1:1 wt%)	CYTOP/Aluminum oxide (Al_2_O_3_)/nanolaminate (NL)	OFETs	Sensing applications	[180]
~10^3^	6.2 (p-type), 2 (n-type)	7.3 (p-type), 7.8 (n-type)	Polycrystalline silicon (poly-Si)	Silicon dioxide (SiO_2_)	TFTs	Sensing applications—smart packages, biodegradable health monitoring units, flexible displays, and disposable sensor nodes	[175]
1.7 × 10^6^	218.3	-	Indium oxide (In_2_O_3_) nanowires	Microporous silicon dioxide (M-SiO_2_)	NW-PFETs	Sensing applications—battery-powered-portable sensors	[185]
>10^9^	~6	-	Graphene/molybdenum disulfide (MoS_2_)	Aluminum oxide (Al_2_O_3_)	Monolayer GFETs	Sensing applications—disposable smart wireless nanosystems and sensors	[188]
>10^7^	0.25 ± 0.023	35.73 ± 1.28	Dinaphthothienothiophene (DNTT)	CYTOP	OFETs	Sensing applications	[189]
>10^3^	0.07	-	Zinc oxide (ZnO)	Polymer electrolyte	EGTs	Sensing applications—biosensors, smart packaging, wearable electronics	[190]
46	-	-	PEDOT/PSS	Cellulose-based electrolyte sticker	OECTs	Sensing applications	[191]
-	-	-	Graphene	Solution-soaked paper	GFETs	Biosensors—glucose and ssDNA sensors	[192]
-	-	-	Hybrid FET device—Commercial Si-based MOSFET (MC14007UBCP) on cellulose paper		EGFETs	Biosensors—pH sensors	[170]
10^2^	20	-	Tin selenide (SnSe_2_)	Nickel oxide (NiO)	MISFETs	Temperature–photo switch sensors	[193]
~10^5^	0.22 ± 0.11 (Average)/0.44 (Maximum)	0.021± 0.63	TIPS-pentacene: polystyrene blend	Poly(4-vinylphenol)/HfO_2_	OFETs	Optical light sensors	[194]
-	-	-	Pencil graphite	Cellulose filter paper	Carbon-based PFETs	Strain sensors (human motion detection)	[173]
99	18.7	-	Graphene/molybdenum disulfide (Gr/MoS_2_)	Cellulose paper	Gr/MoS_2_ PFETs	Strain sensors (human motion detection)	[172]
>10^4^	9.1	1.8	Indium gallium zinc oxide (IGZO)	Ion-gel/cellulose fiber composite dielectric	TFTs	Tactile sensors; digital circuits—inverter, multiplexed active-matrix arrays	[195]

**Table 5:** PFETs for analog/digital circuits and memories.

I_on_/I_off_	Carier Mobility (cm^2^V^−1^S^−1^)	Threshold Voltage (V_TH_) (V)	Semiconductor	Dielectric	Type of Device	Applications	Ref.
200 ± 130	0.086 ± 0.003	0.7 ± 0.1	FS-0027 organic semiconductor ink (PTAA (Poly(3-hexylthiophene-2,5-diyl))-based)	Amorphous silica–MMAcoMAA	TFTs	Digital circuits	[197]
-	-	-	Single-walled carbon nanotubes (SWCNTs)	Poly(methyl methacrylate) (PMMA)	SWCNT FET	Digital circuits—logic gates	[198]
10^4^	21.7 ± 2.70	-	Zinc oxide (ZnO)/carboxymethyl cellulose (CMC)	Cellulose-based ionic conductive hydrogel (CICH) sticker	EGTs	Digital circuits—logic gates	[47]
1.8 × 10^7^	42	0.79	Indium gallium zinc oxide (IGZO)	Graphene oxide-enhanced poly(vinyl alcohol) (PVA)	TFTs	Digital circuits—resistor loaded inverter	[199]
~10^4^	0.56(±0.16)	0.56(±0.24)	TIPS-pentacene/polystyrene blend	Bilayer dielectric (HfO2/PVA)	OFETs	Digital circuits—resistor loaded inverter	[200]
7200	2.6 × 10^−3^	0.6	Poly(3-hexylthiophene) (P3HT) blended with Poly(L-lactic acid) (PLLA) (20:80 wt%)	Deep eutectic mixture of sorbitol + choline chloride (CSorb) solidified with a commercial water-based binder	IMTs	Digital circuits—logic gates, inverters, ring oscillators and memories	[201]
10^7^ (p-type), 4 × 10^6^ (n-type)	1.12 (p-type), 0.17 (n-type)	1.4 (p-type)	Dinaphtho[2,3-b:2′,3′-f]thieno[3,2-b]thiophene (DNTT) (p-type)/ Polyera ActivInk N1100 (PTCDI derivative) (n-type)	Aluminum oxide and alkyl or fluoroalkylphosphonic acid self-assembled monolayer (SAM)	TFTs	Digital circuits—unipolar and complementary ring oscillators	[48]
>10^7^	26	~0.52	Indium zinc oxide (IZO)	Cellulose nanofiber (CNF) electrolyte with EDL capacitance	EDL-TFTs	Digital circuits—inverters, logic gates	[202]
>10^4^	~2	-	Indium gallium zinc oxide (IGZO)	Paper (cellulose-based)	MISFETs	Digital circuits—logic gates	[203]
7.5 × 10⁶	7.8	0.32	Indium tin oxide (ITO)	Cellulose nanofiber (CNF)-soaked paper	TFTs	Digital circuits—logic gates, inverters	[204]
-	-	-	Indium tin oxide (ITO)	Ionic liquid (1-ethyl-3-methylimidazolium tetrafluoroborate) + chitosan on paper	TFTs	Digital circuits—logic gates, inverters	[205]
5.8 × 10⁶	12.8	−0.53 V to 0.97 V (via secondary gate)	Indium tin oxide (ITO)	Chitosan (solution-processed)	TFTs	Digital circuits—logic gates	[206]
~10⁶	~12.8	~0.1	Indium tin oxide (ITO)	Egg albumen-based biopolymer electrolyte	TFTs	Digital circuits—logic gates, inverters	[207]
~1.1 × 10⁶	~11.5	2.5	Indium gallium zinc oxide (IGZO)	Chicken albumen, enhanced with Al_2_O_3_	TFTs	Transistor memories	[49]
-	~0.92 cm^2^/V·s (max); ~0.71 cm^2^/V·s (average)	-	Pentacene	Cross-linked PVP P(VDF-TrFE-CTFE) AlOx/PMMA	OFETs	Transistor memories	[211]
~3.45 × 10^2^	-	-	P3HT (poly(3-hexylthiophene))	P(VDF-TrFE)	FeFETs	Transistor memories	[212]

**Table 6:** Summary of detection techniques in paper-based sensors.

Technique	Principle	Output	Sensing Application Examples
Resistive/capacitive	Change in impedance upon stimulus	Electrical	Humidity [65], pressure [59], strain [83]
Electrochemical	Redox signal at printed electrodes	Electrical	Glucose [123], VOCs [62]
FET-based sensing	Current modulation via gate-channel field	Electrical	Glucose [192], nucleic acid detection [185]
Colorimetric	Color change by reaction	Optical	Heavy metals [99], pesticides [53]
Fluorescence	Light emission under excitation	Optical	Cancer [145], health [93] biomarkers

**Table 7:** Comparison of paper-based devices.

Device Type	Functionality	Complexity	Fabrication Techniques	Materials Used	Advantages	Limitations
Resistive/Capacitive Sensors	Detect humidity, pressure, strain, temperature	Low	Dip-coating, laser patterning, hand-drawing	Graphene, CNTs, graphite, rGO, cellulose composites	Simple, low-cost, flexible, disposable	Limited sensitivity and selectivity; environmental instability
Optical Sensors	Colorimetric or fluorescence detection of analytes	Medium	Drop casting, printing, nanoparticle deposition	AgNPs, CuNPs, QDs, graphene oxide, dyes	Visual readout, smartphone-compatible, user-friendly	Limited quantification; often single use; dependent on lighting
Electrochemical Sensors	Measure redox reactions for analyte detection	Medium–High	Screen printing, inkjet printing, electrode modification	Carbon inks, metal oxides, conductive polymers	High sensitivity, quantitative, scalable	Requires external reader; sensitive to interference and drift
PFETs (Field-Effect Transistors)	Signal amplification, switching, sensing, logic	High	Printing, vapor deposition, doping, photolithography	Organic semiconductors, ion gels, graphene, PEDOT/PSS	Enable active electronics, logic gates, multifunctionality	Poor mobility/stability; complex fabrication; low integration maturity
